# Supplementary figures and images for: Hundreds of myosin 10s are pushed to the tips of filopodia and could cause traffic jams on actin
Source: eLife. 2024 Oct 31;12:RP90603. doi: 10.7554/eLife.90603 (PMC11527427; doi:10.7554/eLife.90603)

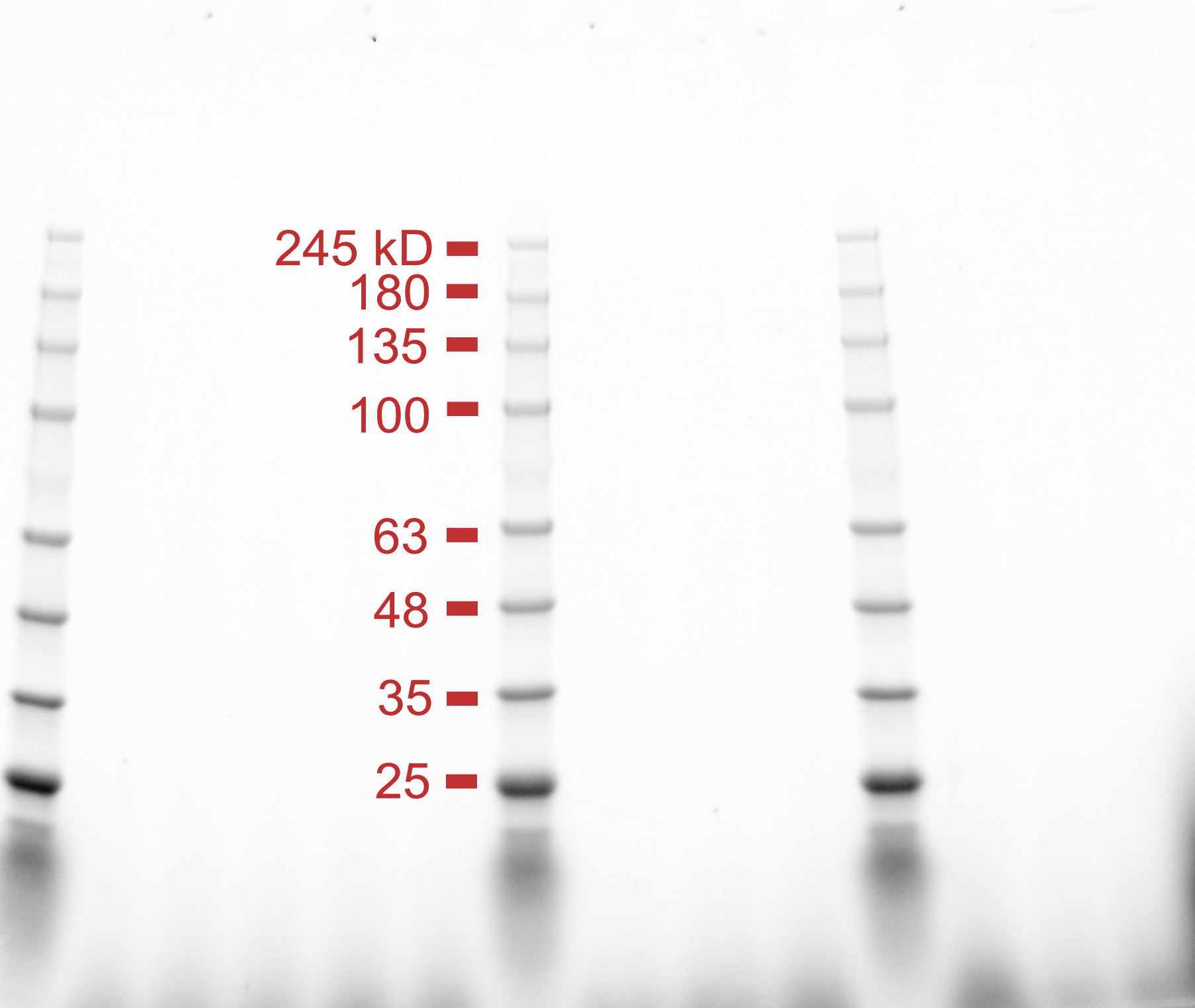

Supplement: Figure 1—source data 1. [file elife-90603-fig1-data1.zip › Figure 1-source data 1/Figure1B_ladder_annotated.tiff]

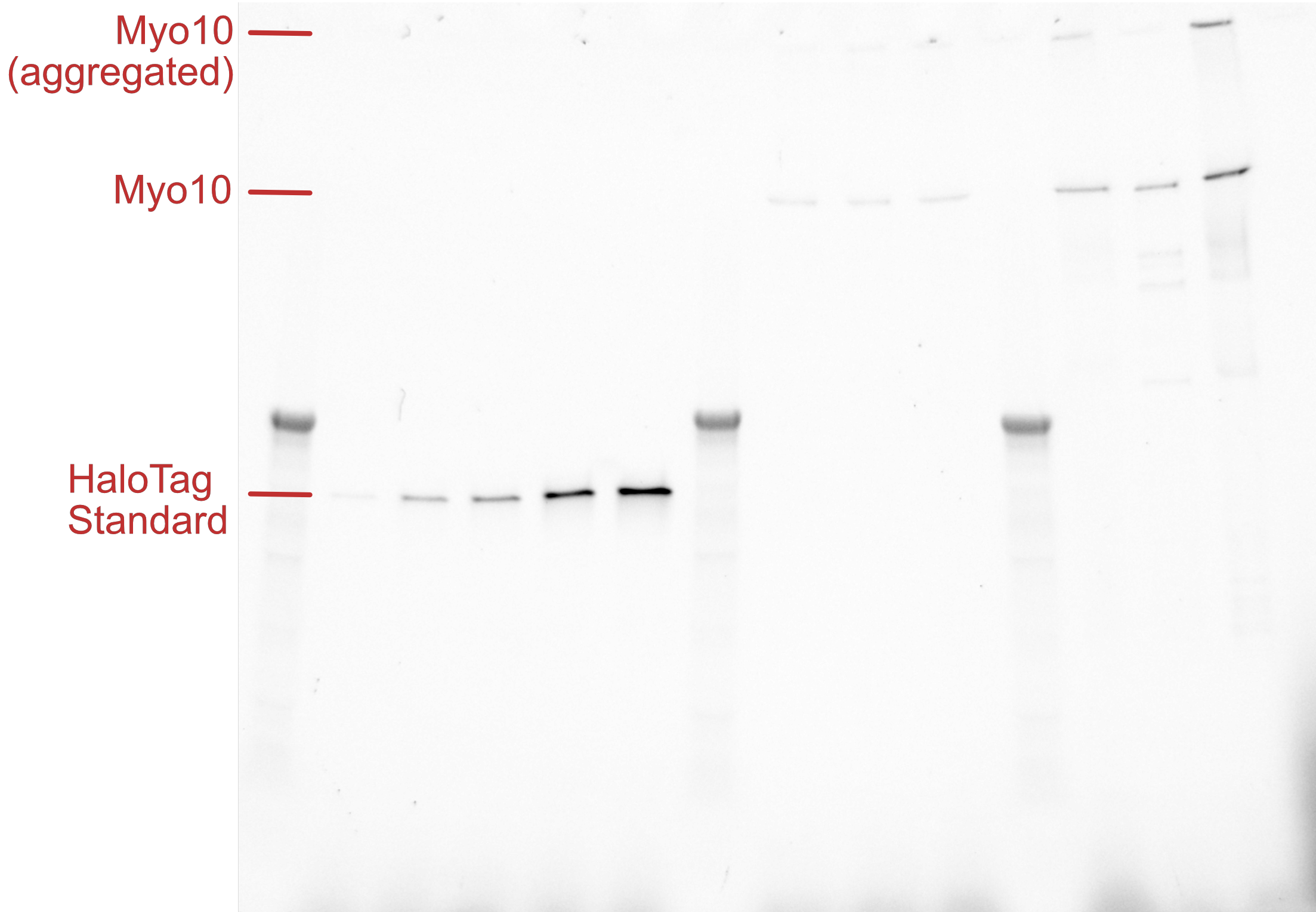

Supplement: Figure 1—source data 1. [file elife-90603-fig1-data1.zip › Figure 1-source data 1/Figure1B_rhodamine_annotated.tiff]

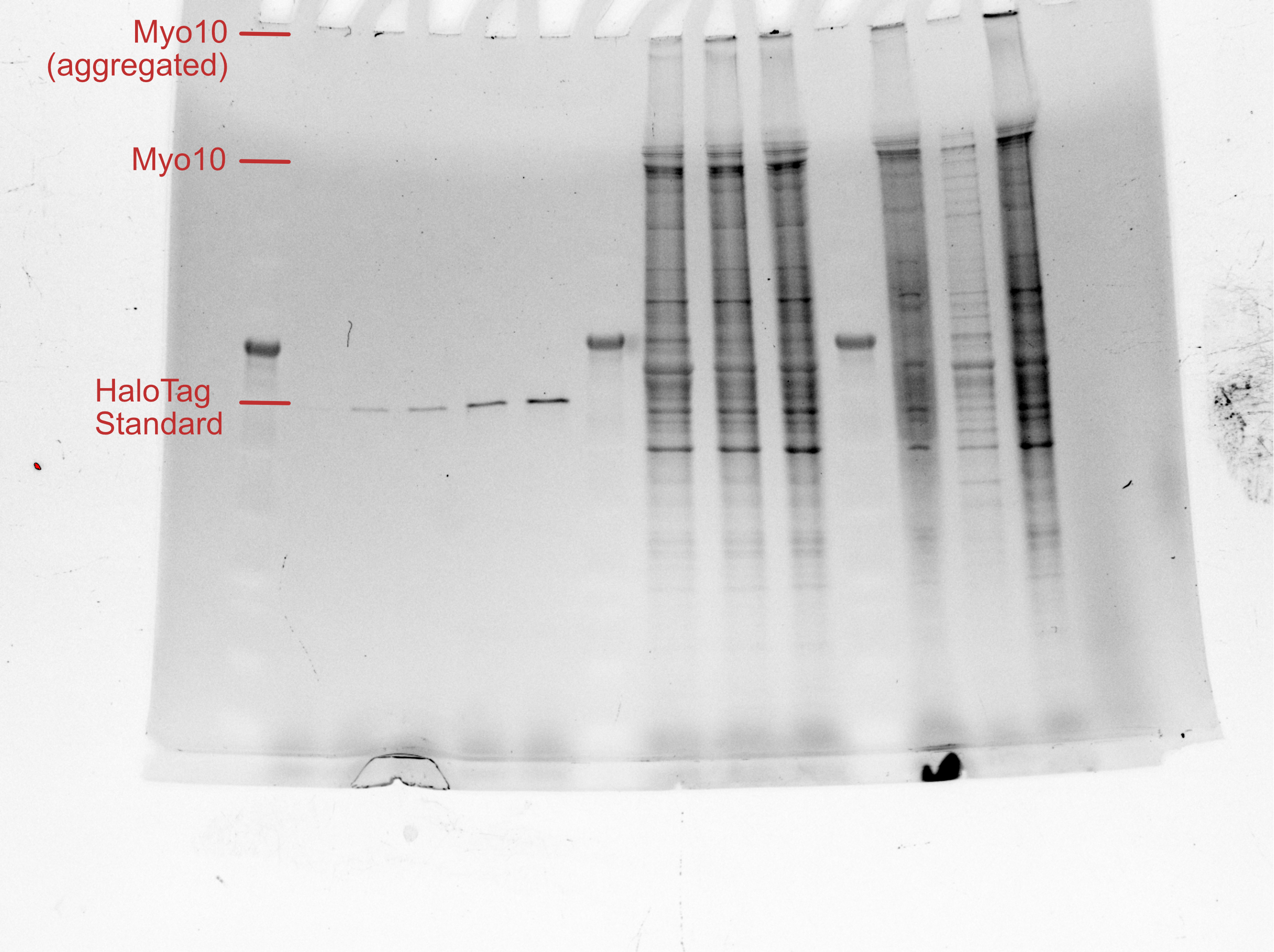

Supplement: Figure 1—source data 1. [file elife-90603-fig1-data1.zip › Figure 1-source data 1/Figure1B_stain_free_protein_annotated.tiff]

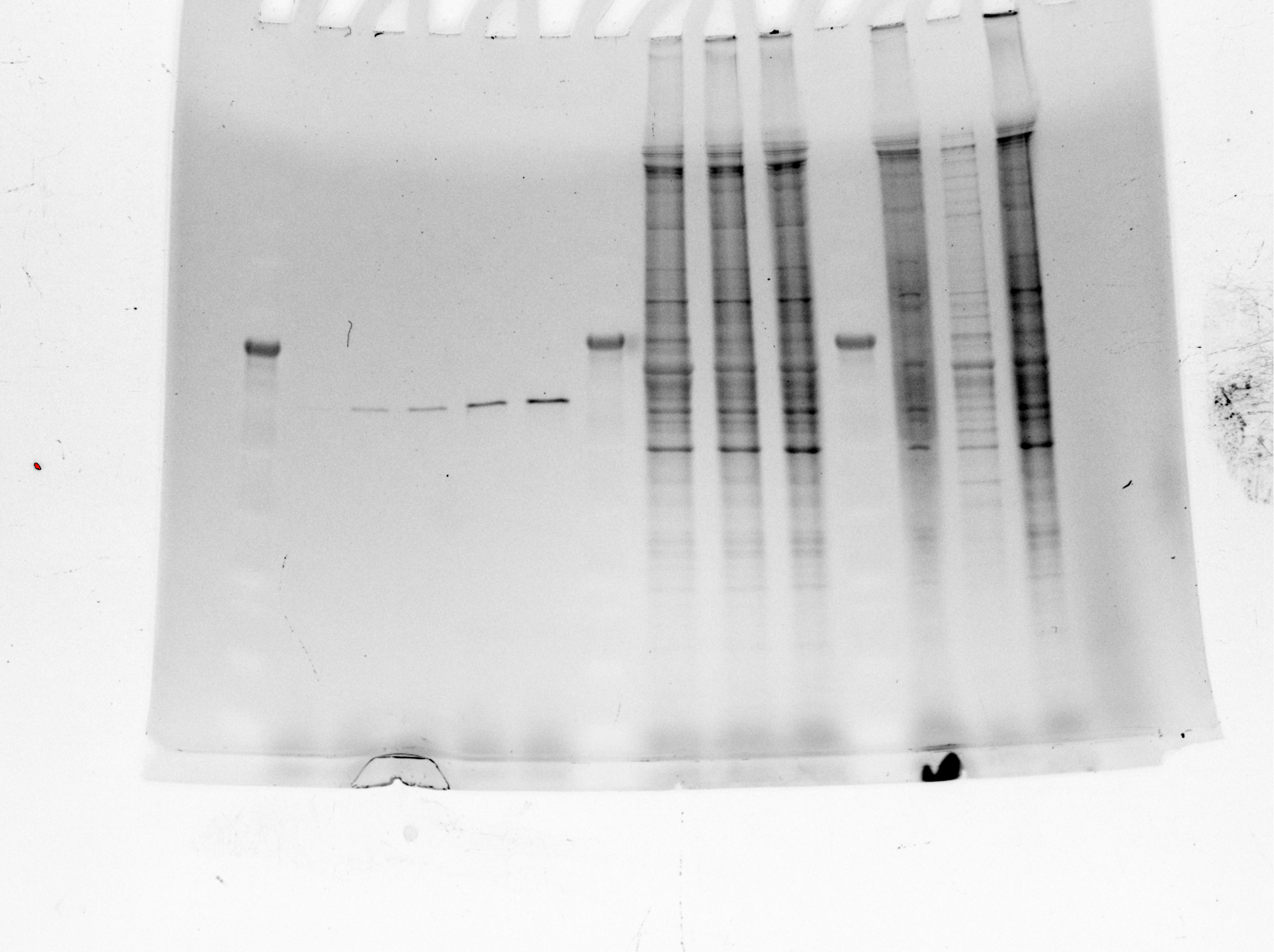

Supplement: Figure 1—source data 2. [file elife-90603-fig1-data2.zip › Figure 1-source data 2/Figure1B_stain_free_protein.tif]

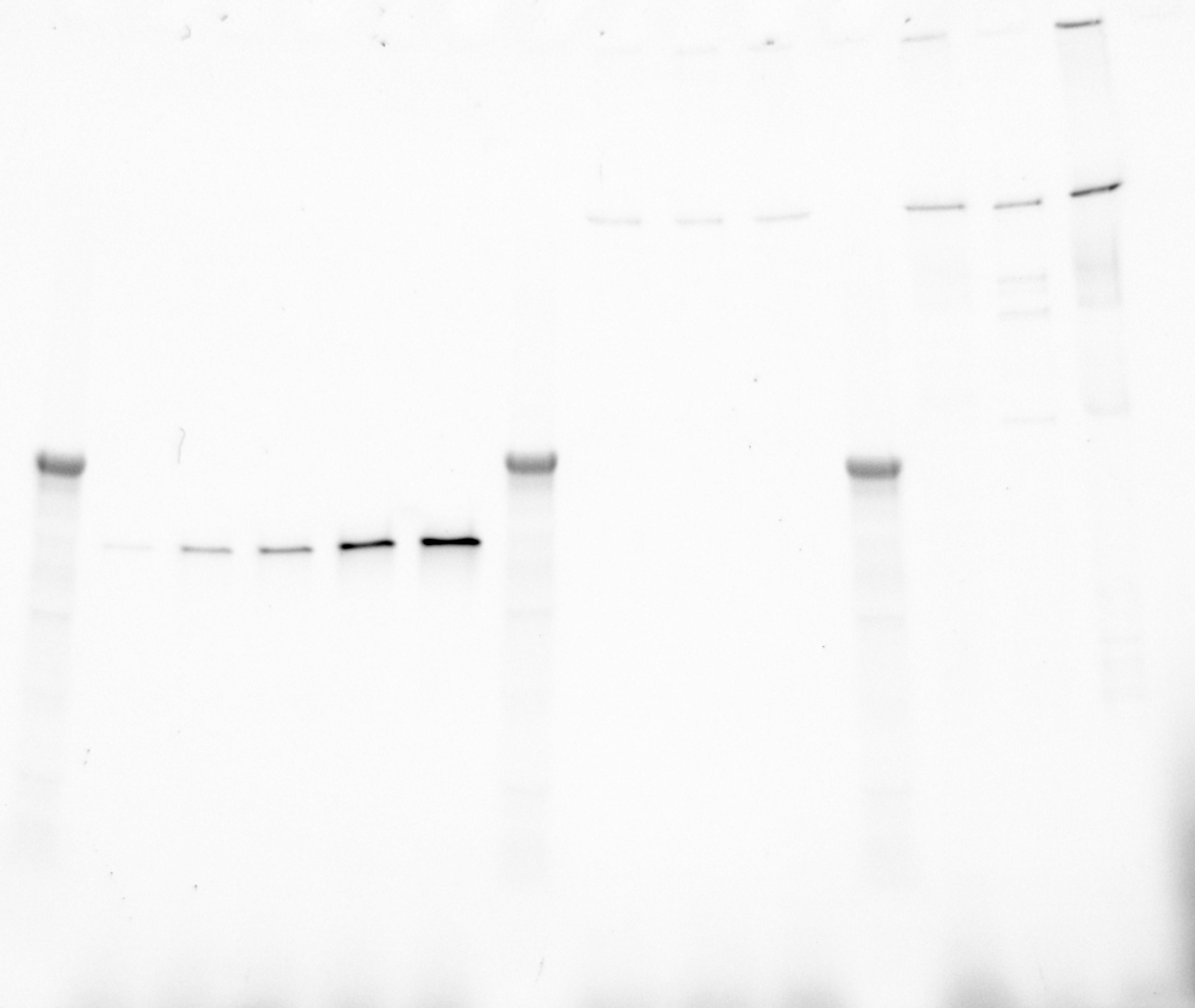

Supplement: Figure 1—source data 2. [file elife-90603-fig1-data2.zip › Figure 1-source data 2/Figure1B_rhodamine.tif]

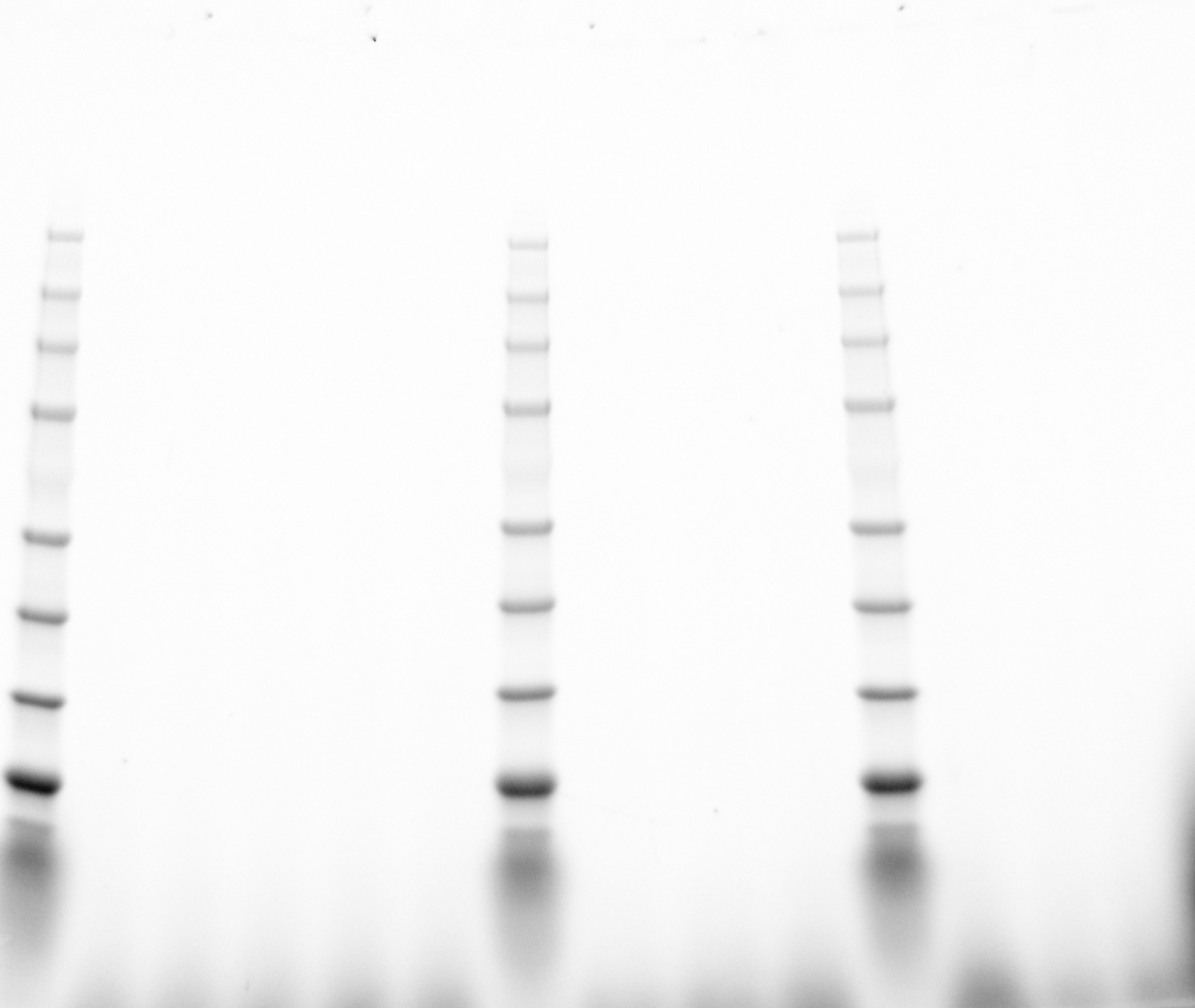

Supplement: Figure 1—source data 2. [file elife-90603-fig1-data2.zip › Figure 1-source data 2/Figure1B_ladder.tif]

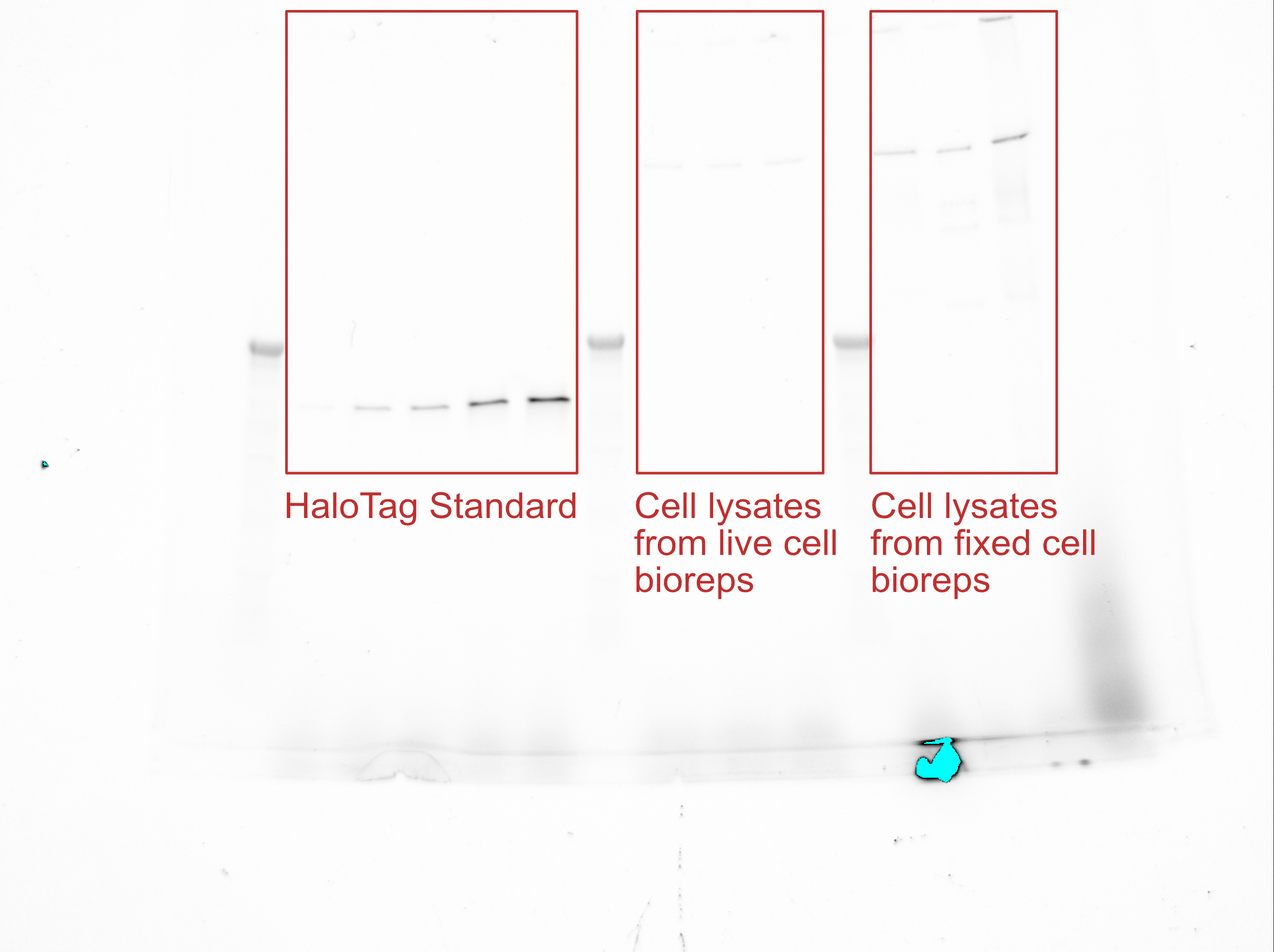

Supplement: Figure 1—figure supplement 1—source data 1. [file elife-90603-fig1-figsupp1-data1.zip › Figure 1ΓÇöfigure supplement 1-source data 1/Figure1-figure supplement 1C_rhodamine_annotated.tiff]

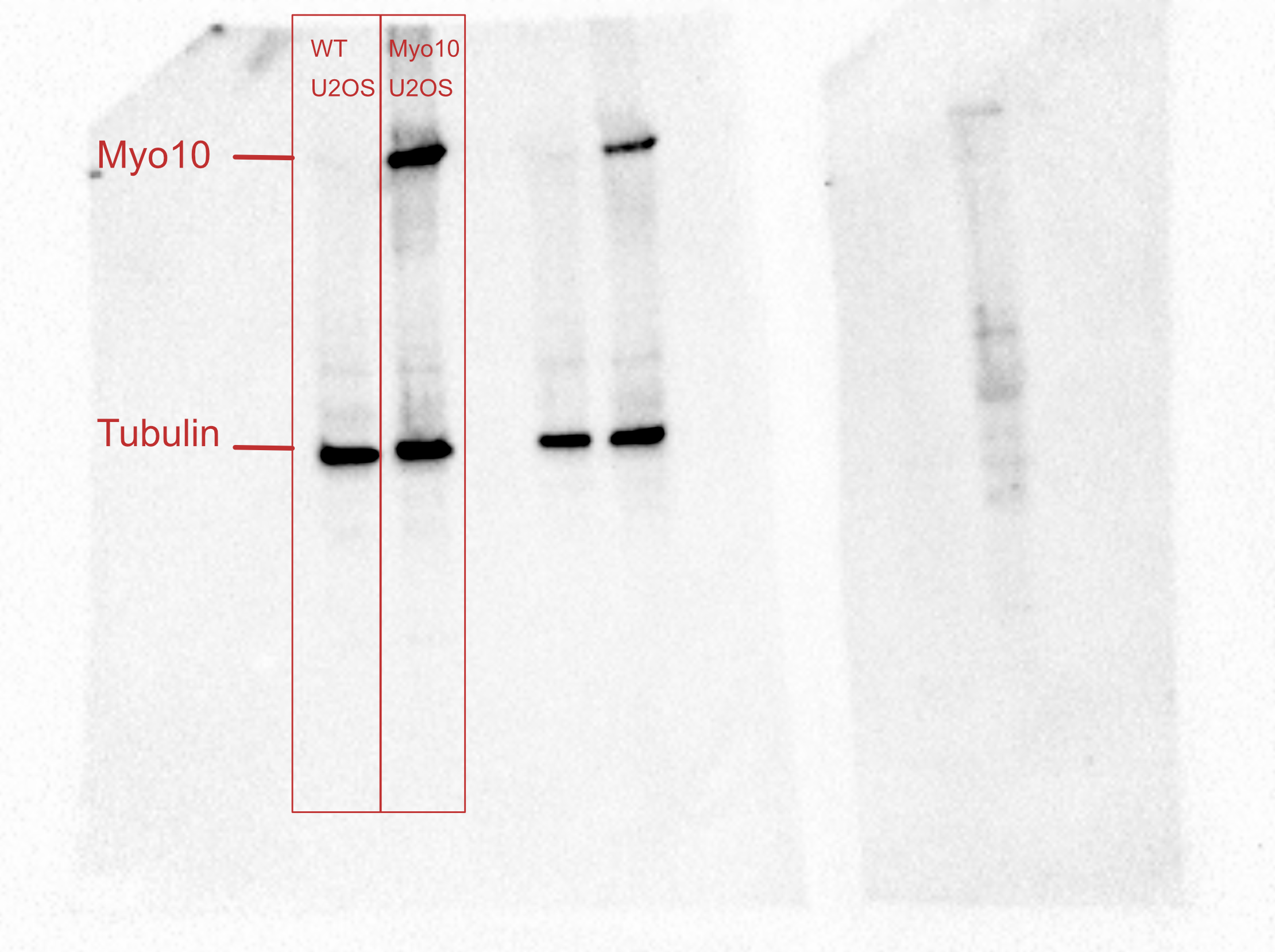

Supplement: Figure 1—figure supplement 1—source data 1. [file elife-90603-fig1-figsupp1-data1.zip › Figure 1ΓÇöfigure supplement 1-source data 1/Figure 1ΓÇöfigure supplement 1E_blot_annotated.tiff]

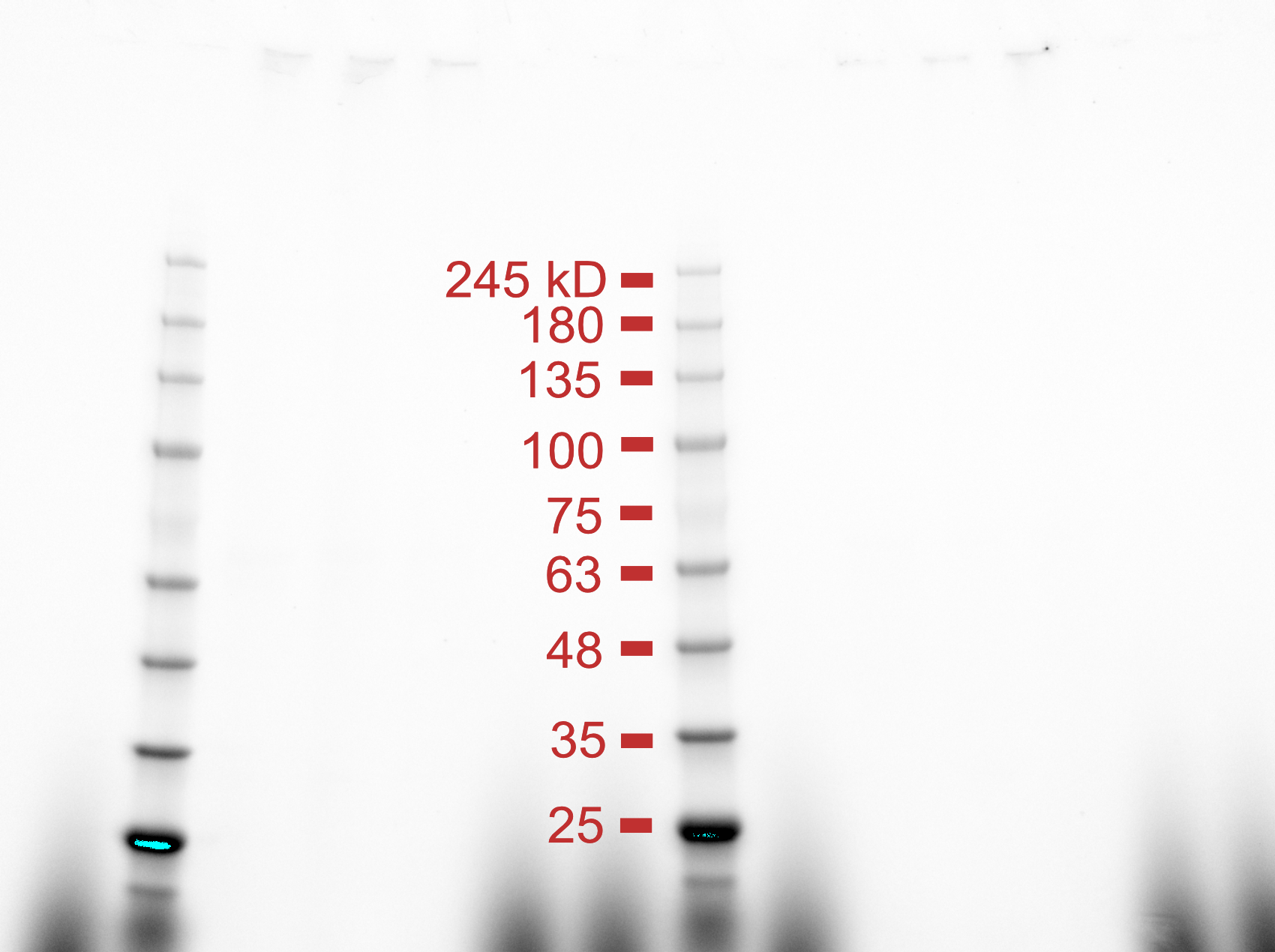

Supplement: Figure 1—figure supplement 1—source data 1. [file elife-90603-fig1-figsupp1-data1.zip › Figure 1ΓÇöfigure supplement 1-source data 1/Figure 1ΓÇöfigure supplement 1D_ladder_annotated.tiff]

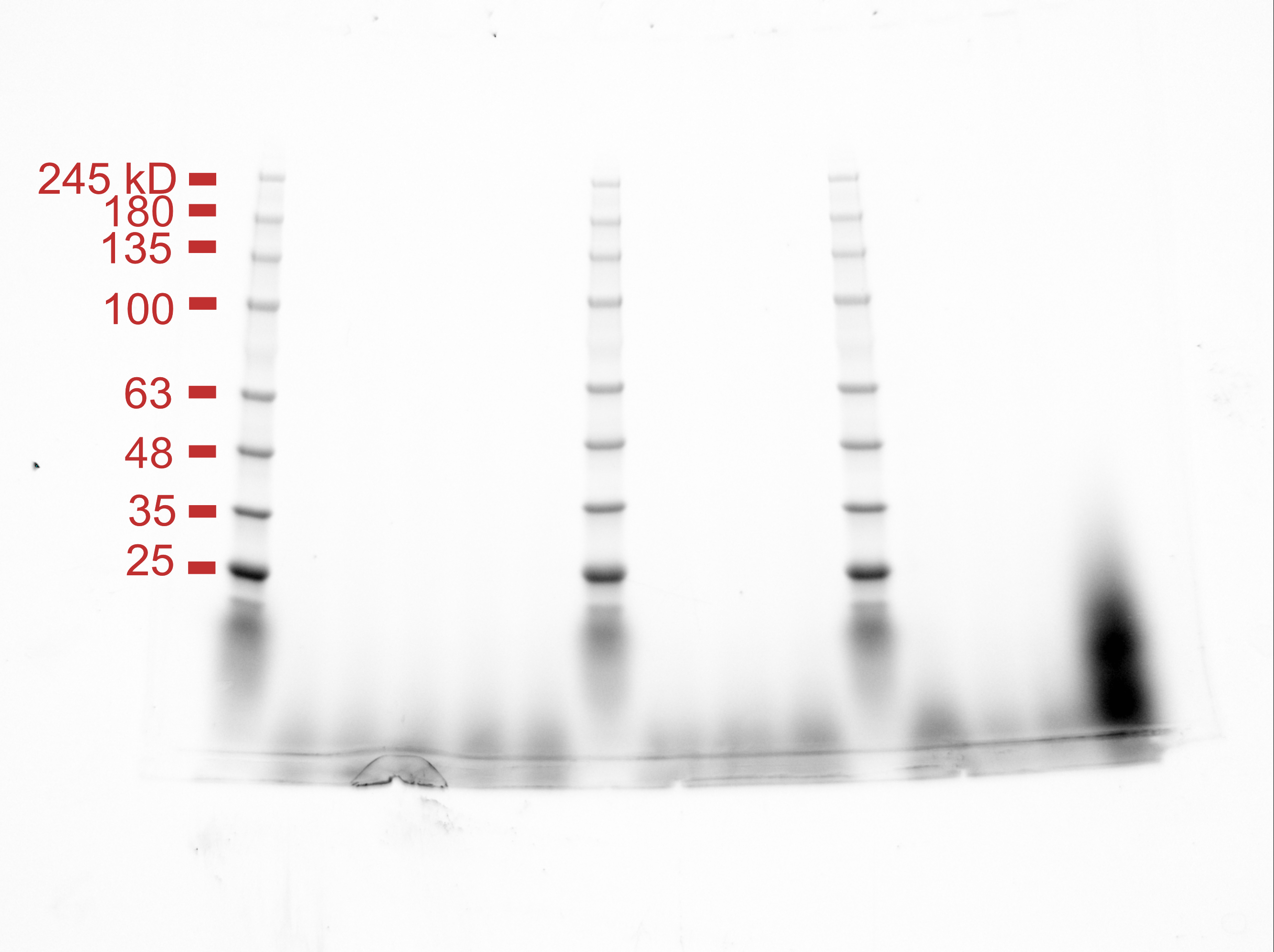

Supplement: Figure 1—figure supplement 1—source data 1. [file elife-90603-fig1-figsupp1-data1.zip › Figure 1ΓÇöfigure supplement 1-source data 1/Figure1-figure supplement 1C_ladder_annotated.tiff]

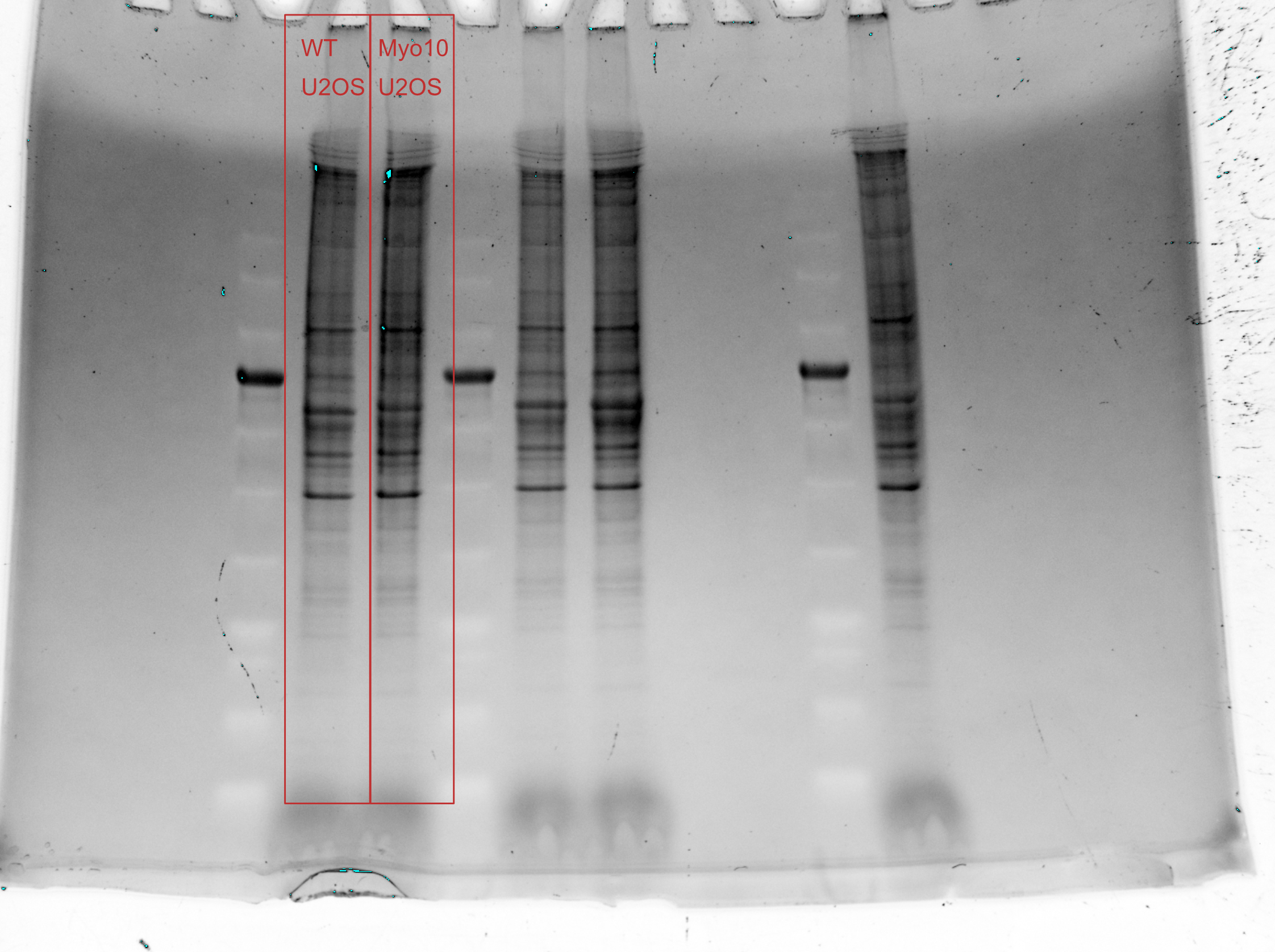

Supplement: Figure 1—figure supplement 1—source data 1. [file elife-90603-fig1-figsupp1-data1.zip › Figure 1ΓÇöfigure supplement 1-source data 1/Figure 1ΓÇöfigure supplement 1E_stain_free_protein_annotated.tiff]

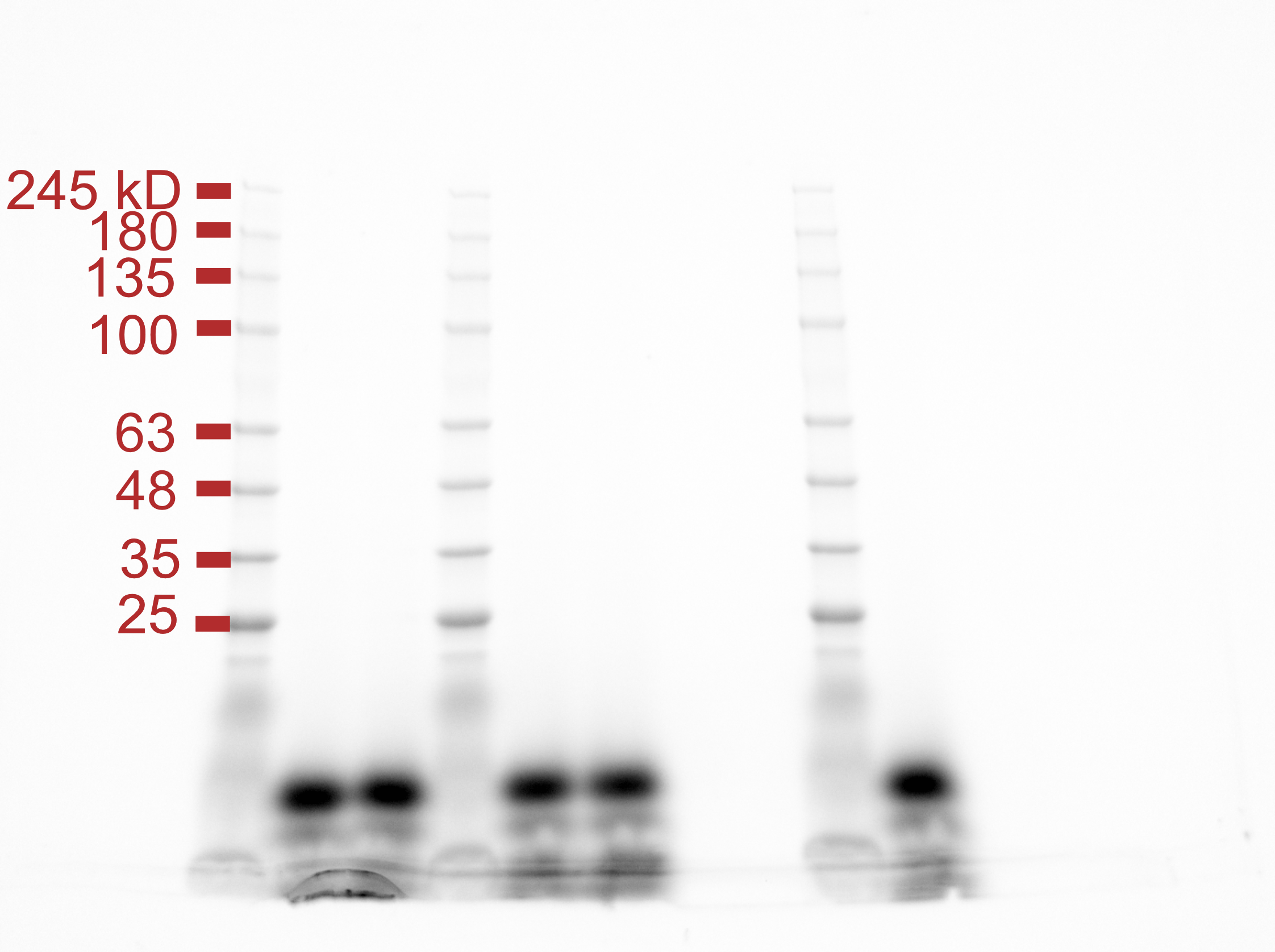

Supplement: Figure 1—figure supplement 1—source data 1. [file elife-90603-fig1-figsupp1-data1.zip › Figure 1ΓÇöfigure supplement 1-source data 1/Figure 1ΓÇöfigure supplement 1E_ladder_annotated.tiff]

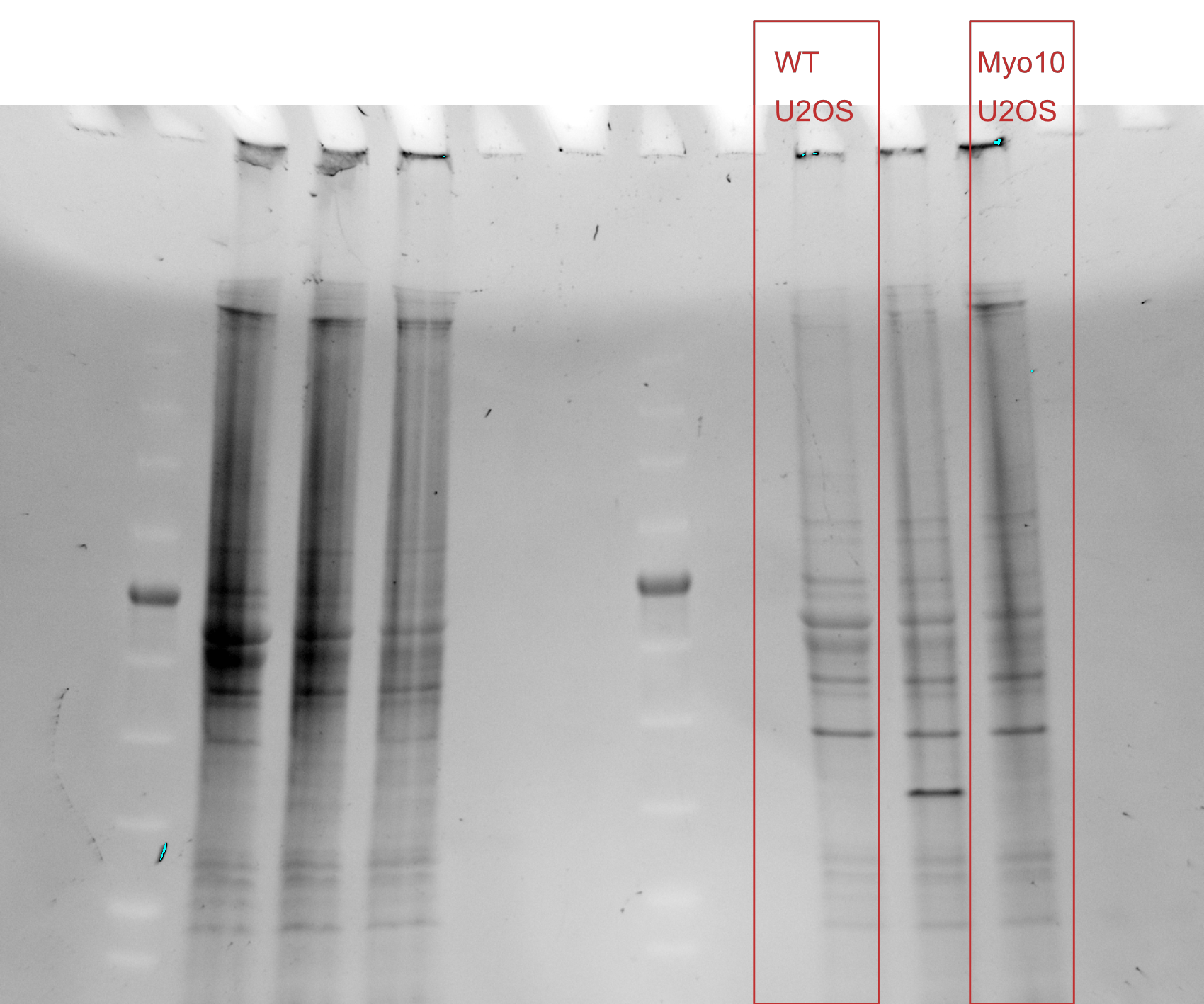

Supplement: Figure 1—figure supplement 1—source data 1. [file elife-90603-fig1-figsupp1-data1.zip › Figure 1ΓÇöfigure supplement 1-source data 1/Figure 1ΓÇöfigure supplement 1D_stain_free_protein_annotated.tiff]

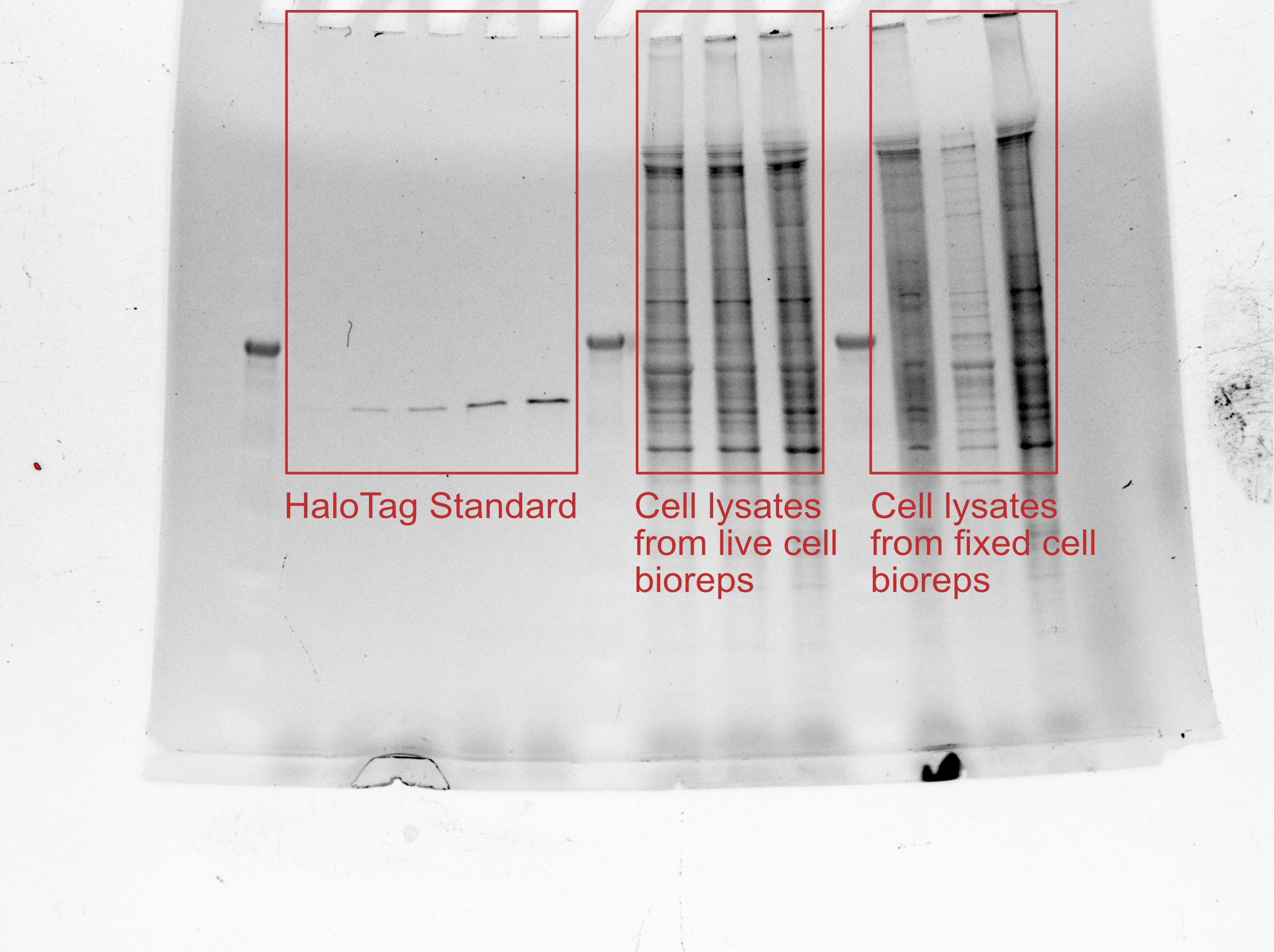

Supplement: Figure 1—figure supplement 1—source data 1. [file elife-90603-fig1-figsupp1-data1.zip › Figure 1ΓÇöfigure supplement 1-source data 1/Figure1-figure supplement 1C_stain_free_protein_annotated.tiff]

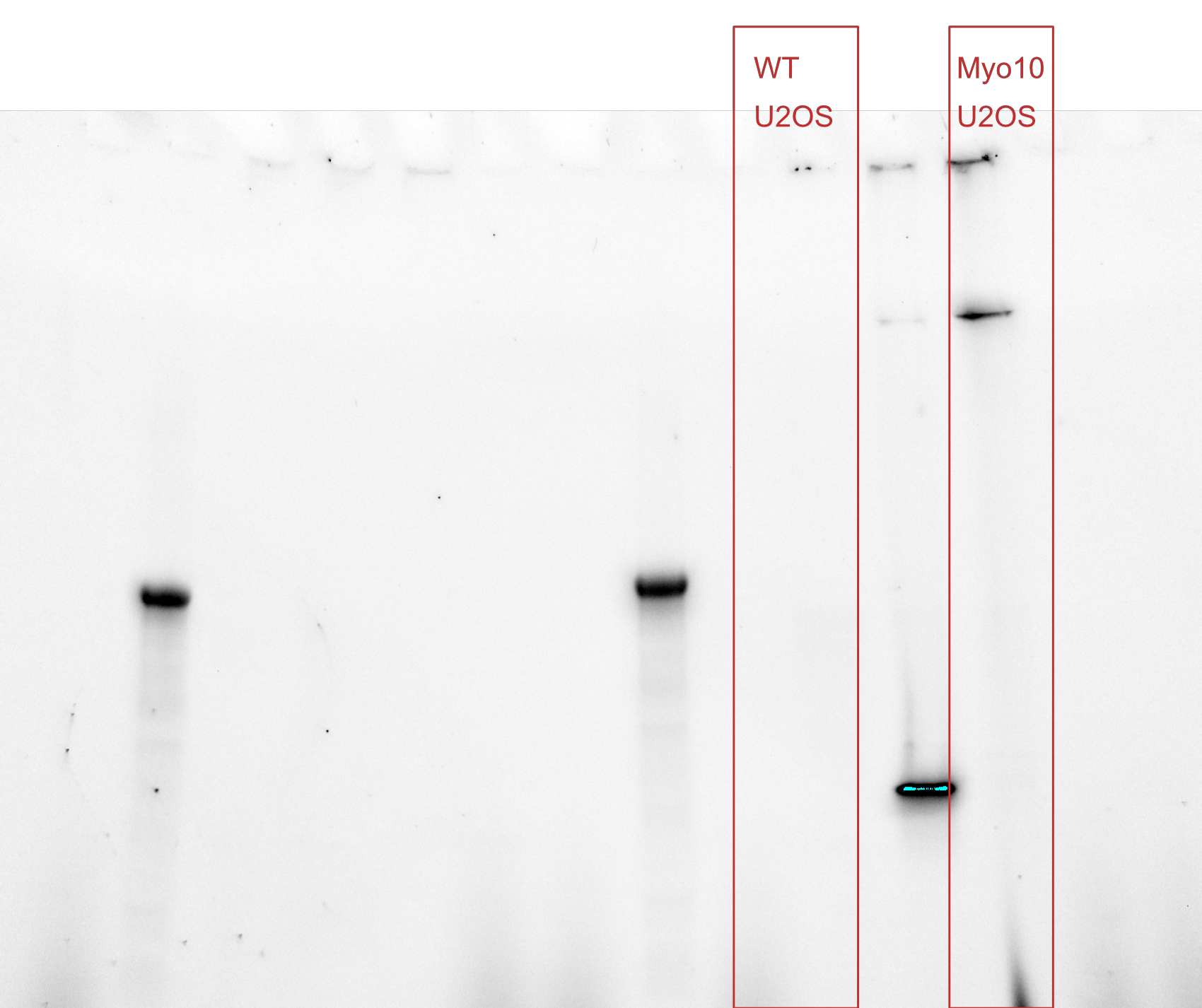

Supplement: Figure 1—figure supplement 1—source data 1. [file elife-90603-fig1-figsupp1-data1.zip › Figure 1ΓÇöfigure supplement 1-source data 1/Figure 1ΓÇöfigure supplement 1D_rhodamine_annotated.tiff]

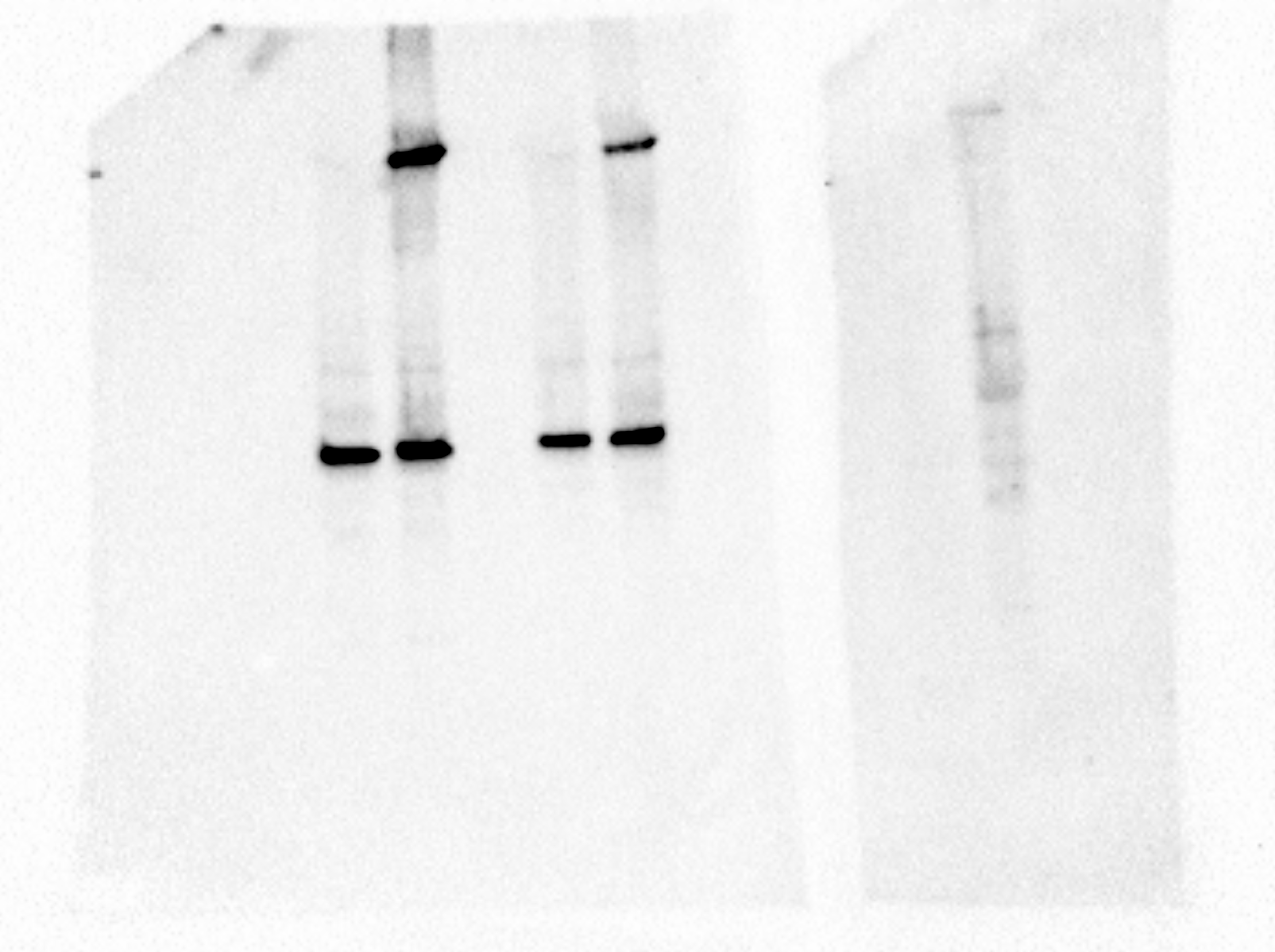

Supplement: Figure 1—figure supplement 1—source data 2. [file elife-90603-fig1-figsupp1-data2.zip › Figure 1ΓÇöfigure supplement 1-source data 2/Figure 1ΓÇöfigure supplement 1E_blot.tiff]

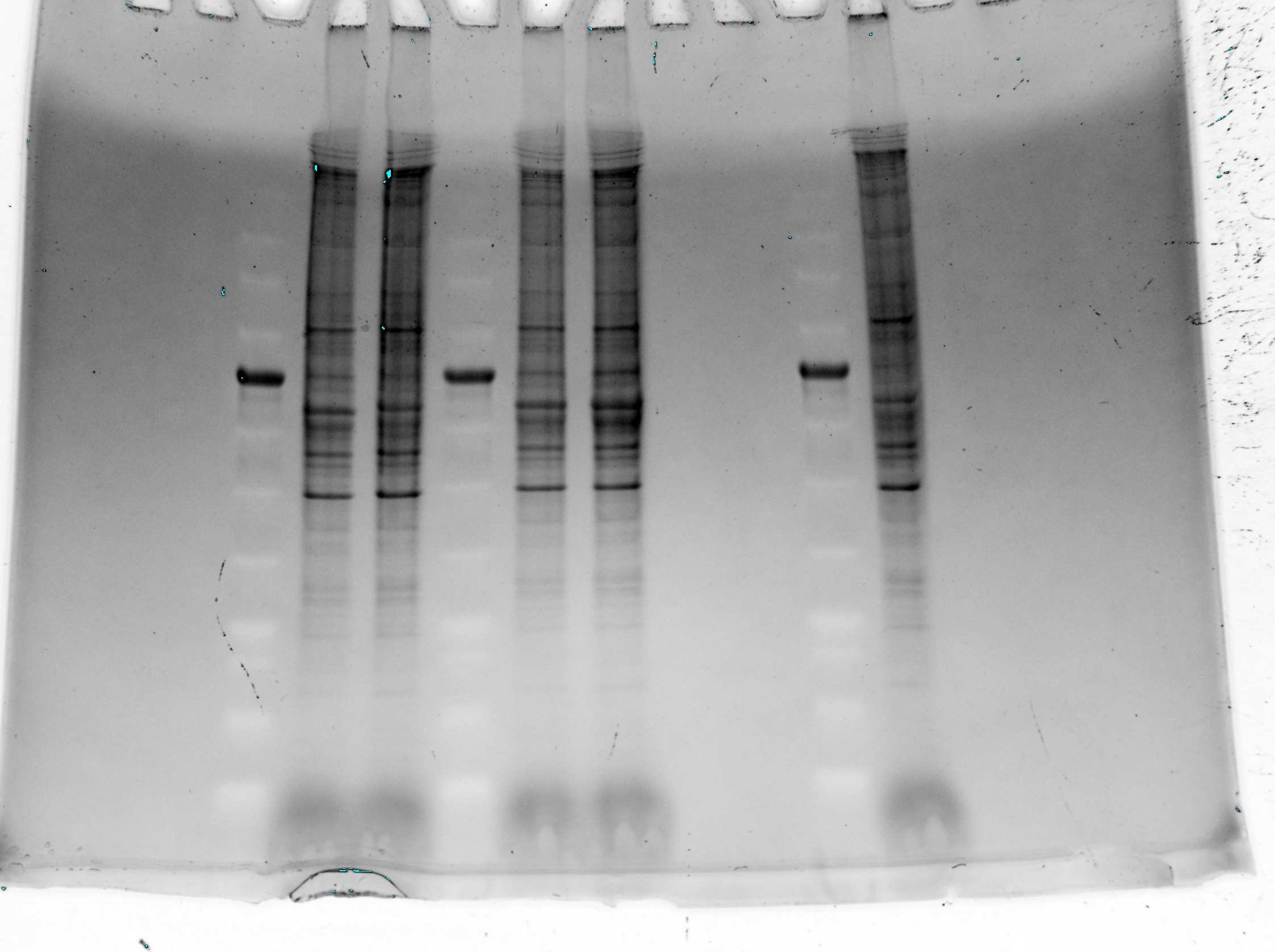

Supplement: Figure 1—figure supplement 1—source data 2. [file elife-90603-fig1-figsupp1-data2.zip › Figure 1ΓÇöfigure supplement 1-source data 2/Figure 1ΓÇöfigure supplement 1E_stain_free_protein.tif]

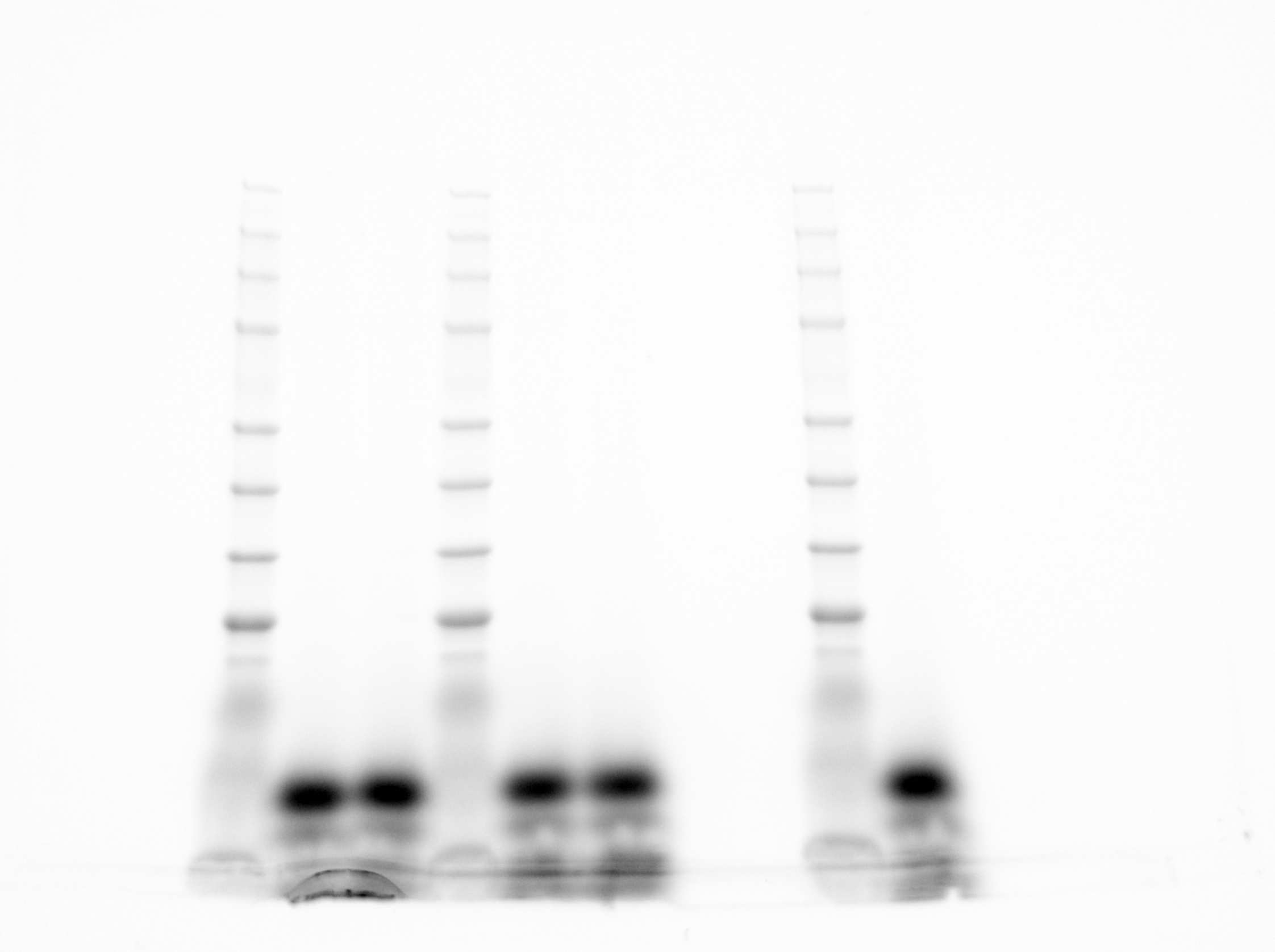

Supplement: Figure 1—figure supplement 1—source data 2. [file elife-90603-fig1-figsupp1-data2.zip › Figure 1ΓÇöfigure supplement 1-source data 2/Figure 1ΓÇöfigure supplement 1E_ladder.tif]

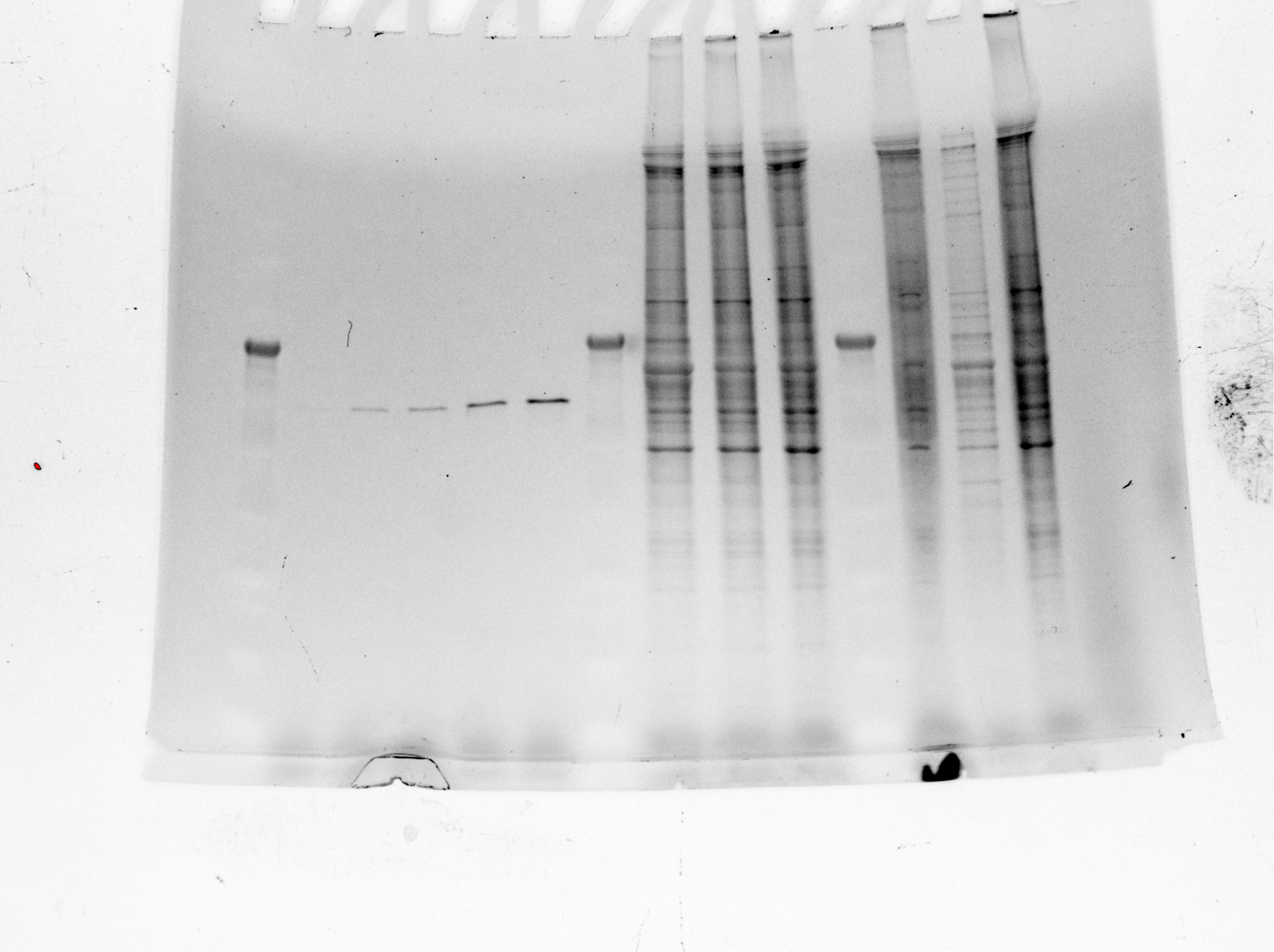

Supplement: Figure 1—figure supplement 1—source data 2. [file elife-90603-fig1-figsupp1-data2.zip › Figure 1ΓÇöfigure supplement 1-source data 2/Figure1-figure supplement 1C_stain_free_protein.tif]

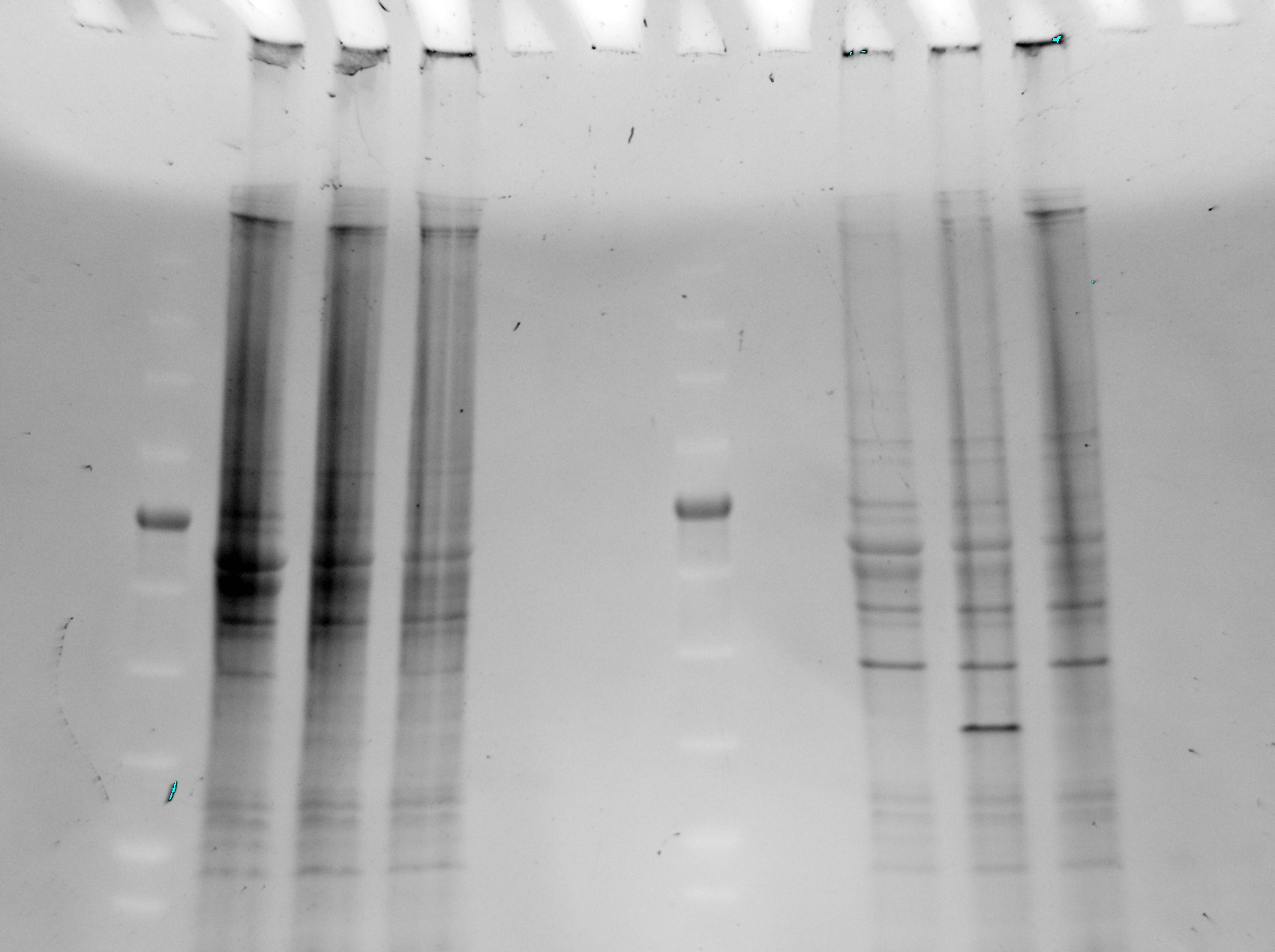

Supplement: Figure 1—figure supplement 1—source data 2. [file elife-90603-fig1-figsupp1-data2.zip › Figure 1ΓÇöfigure supplement 1-source data 2/Figure 1ΓÇöfigure supplement 1D_stain_free_protein.tif]

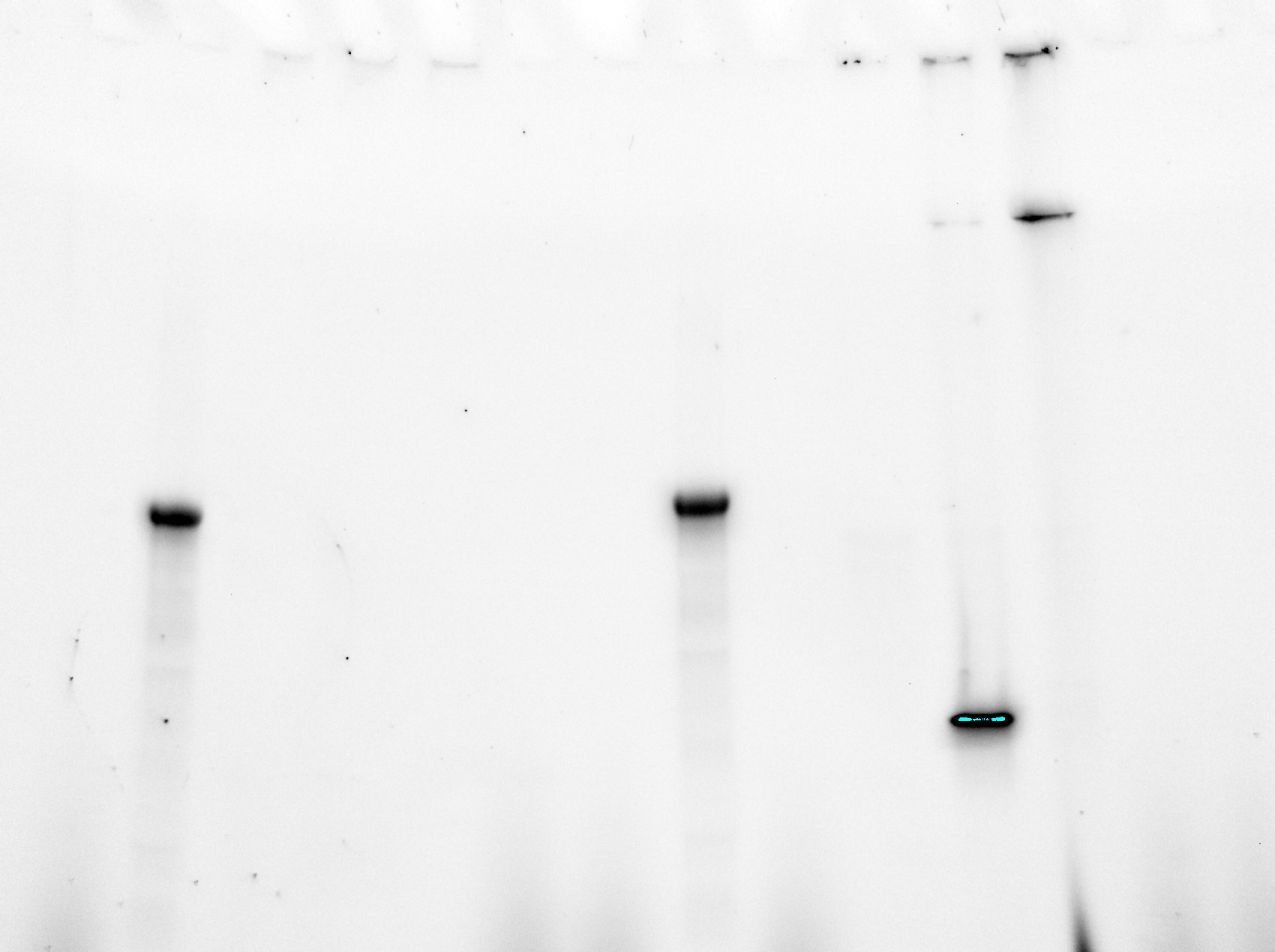

Supplement: Figure 1—figure supplement 1—source data 2. [file elife-90603-fig1-figsupp1-data2.zip › Figure 1ΓÇöfigure supplement 1-source data 2/Figure 1ΓÇöfigure supplement 1D_rhodamine.tif]

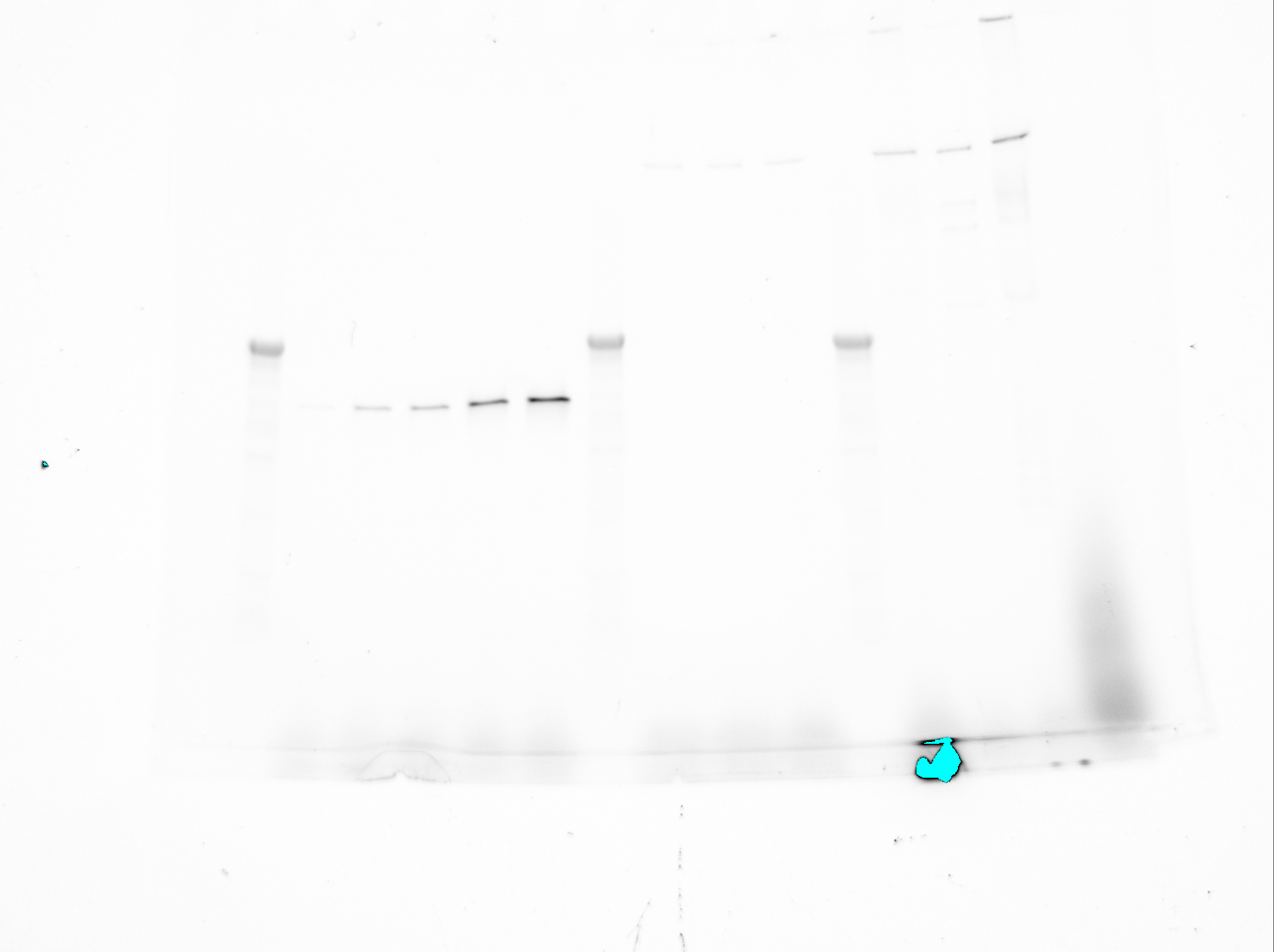

Supplement: Figure 1—figure supplement 1—source data 2. [file elife-90603-fig1-figsupp1-data2.zip › Figure 1ΓÇöfigure supplement 1-source data 2/Figure1-figure supplement 1C_rhodamine.tif]

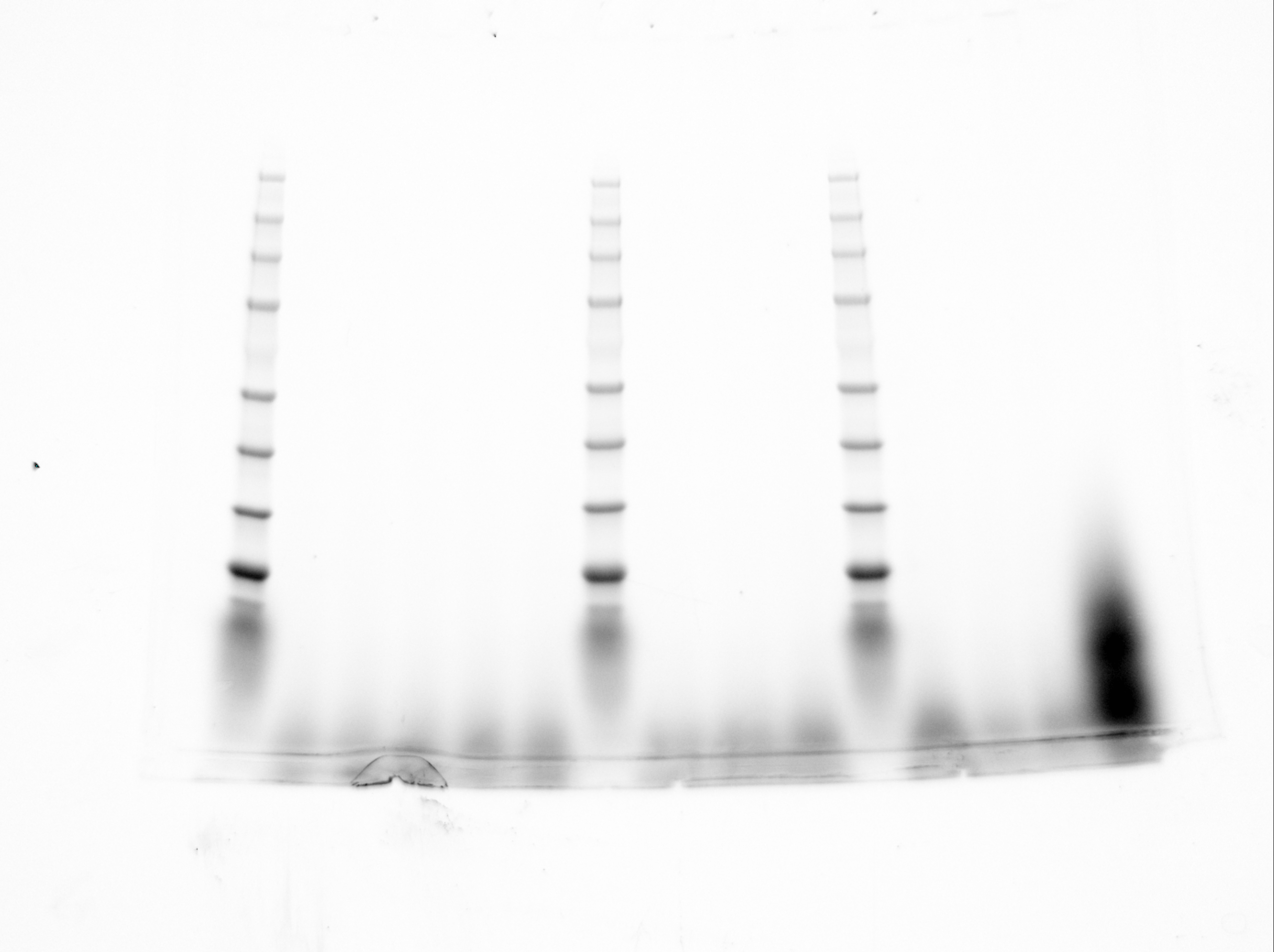

Supplement: Figure 1—figure supplement 1—source data 2. [file elife-90603-fig1-figsupp1-data2.zip › Figure 1ΓÇöfigure supplement 1-source data 2/Figure1-figure supplement 1C_ladder.tif]

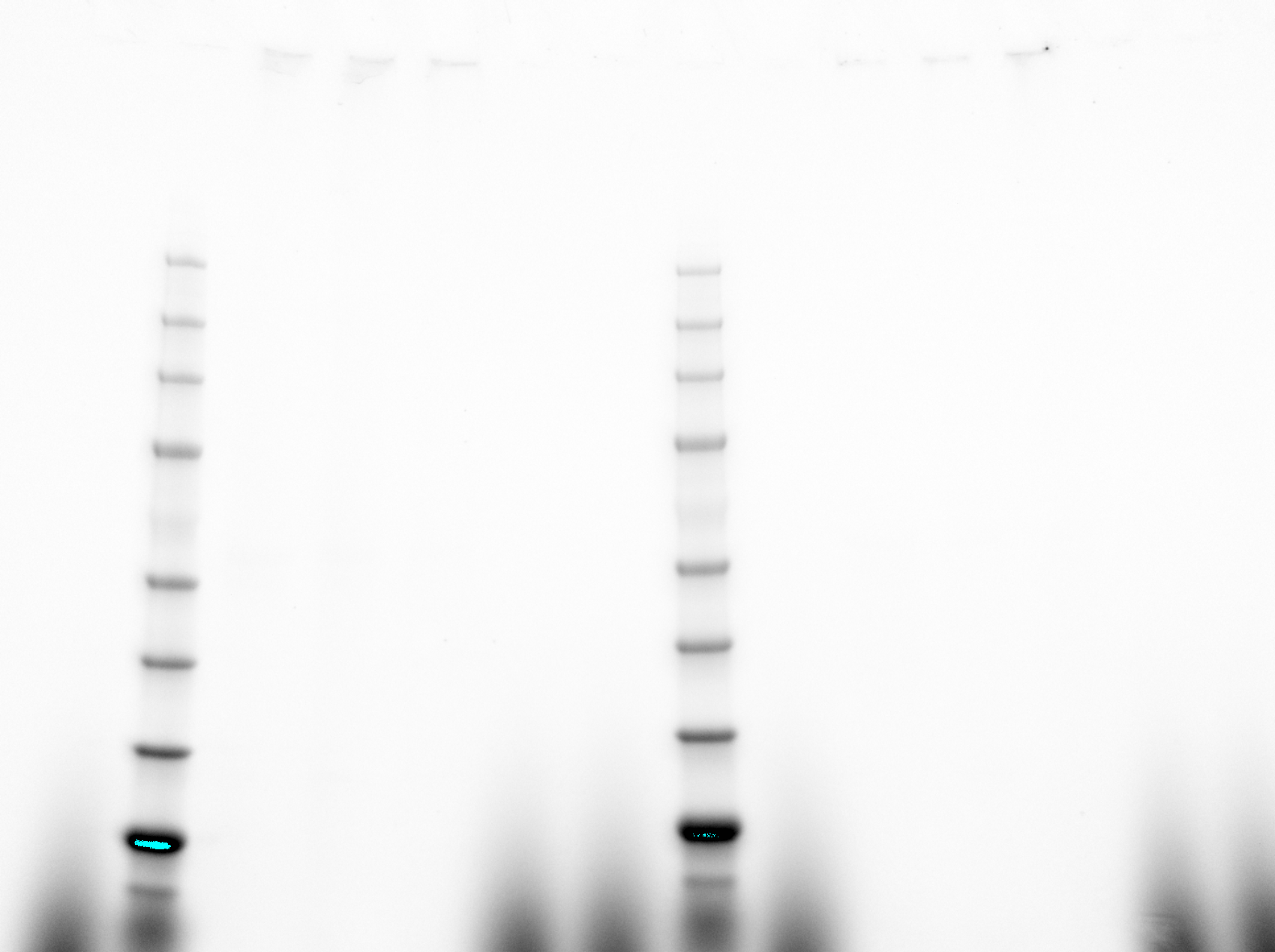

Supplement: Figure 1—figure supplement 1—source data 2. [file elife-90603-fig1-figsupp1-data2.zip › Figure 1ΓÇöfigure supplement 1-source data 2/Figure 1ΓÇöfigure supplement 1D_ladder.tif]

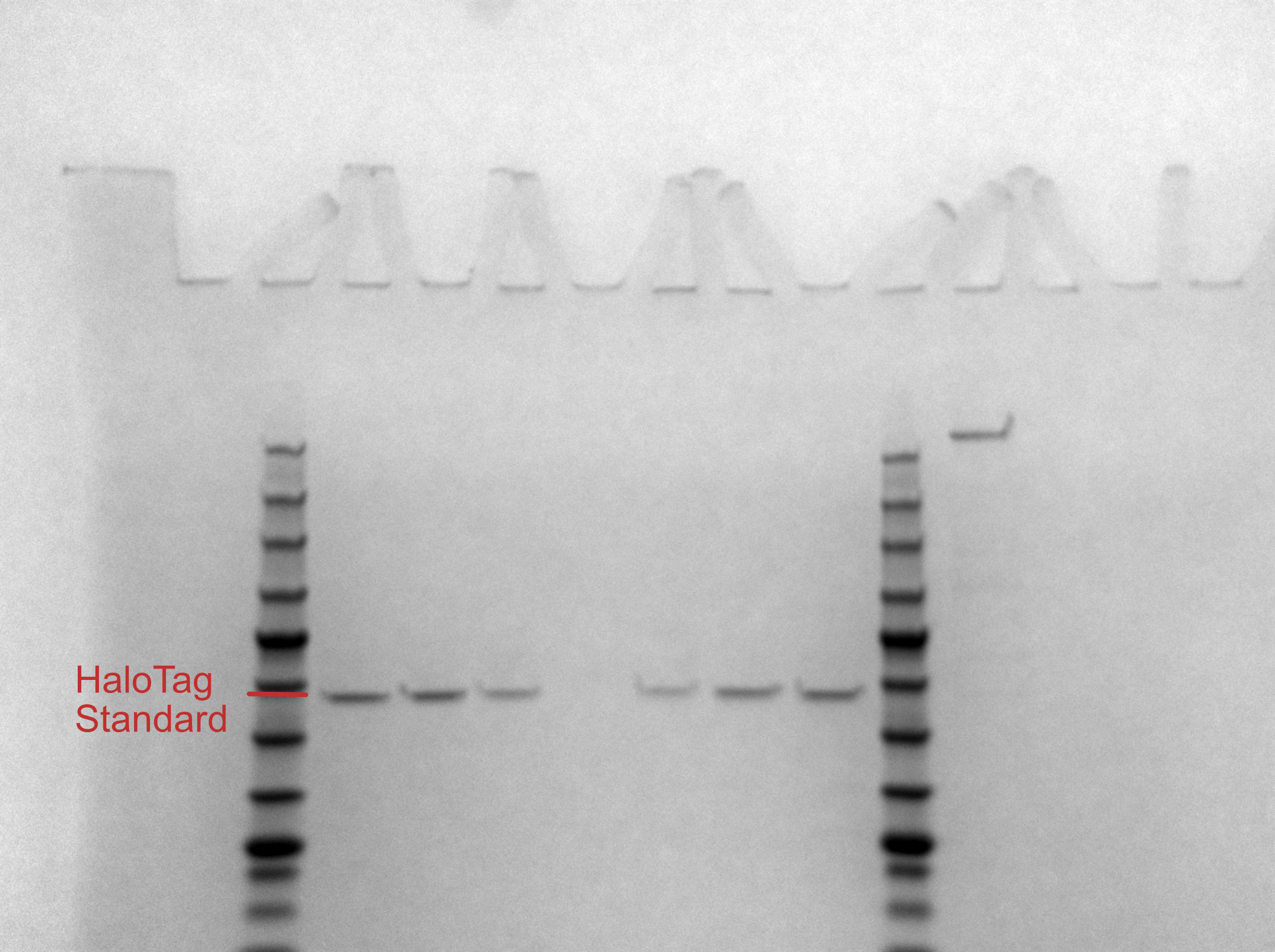

Supplement: Figure 1—figure supplement 2—source data 1. [file elife-90603-fig1-figsupp2-data1.zip › Figure 1ΓÇöfigure supplement 2-source data 1/Figure 1ΓÇöfigure supplement 2A_coomassie_annotated.tiff]

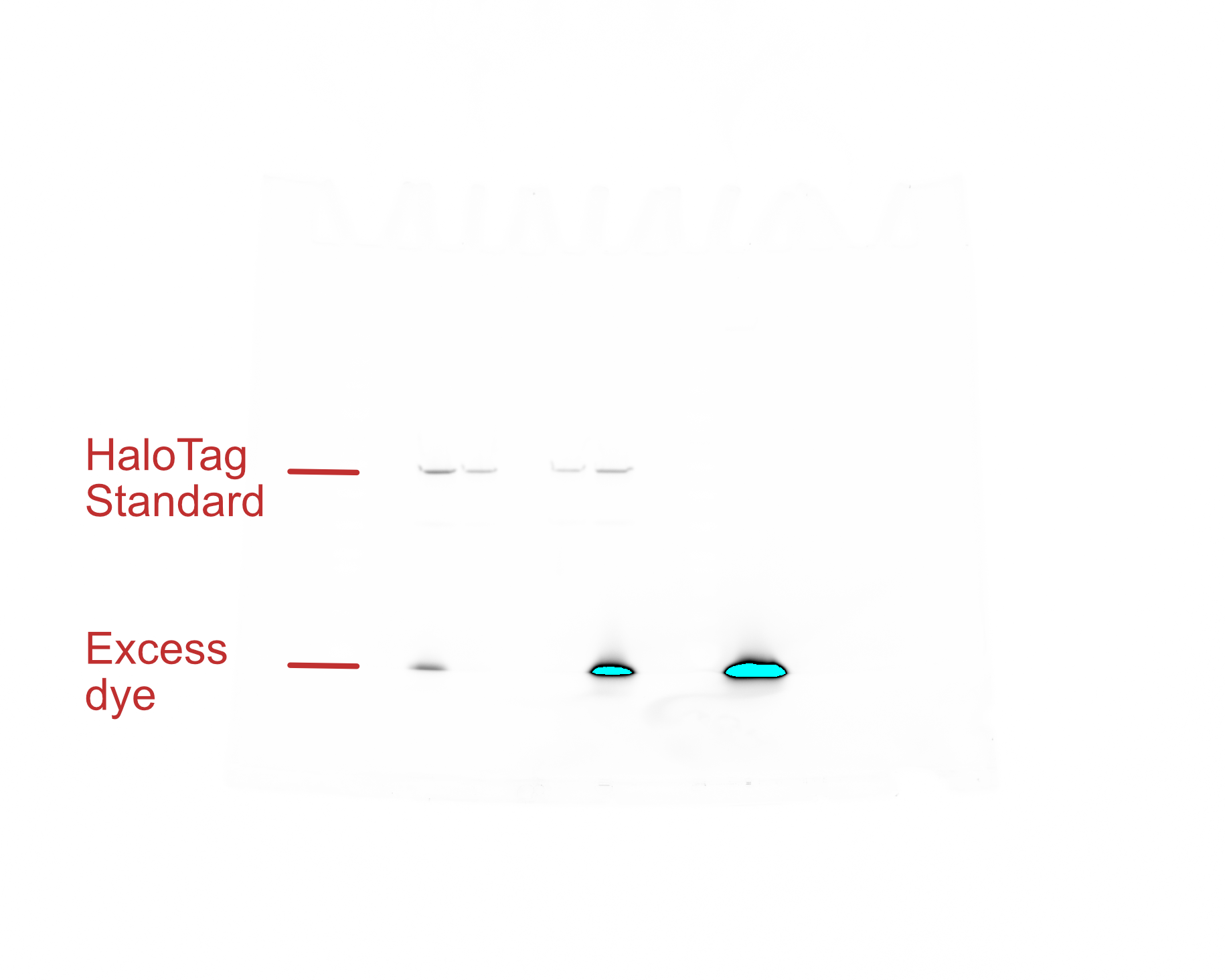

Supplement: Figure 1—figure supplement 2—source data 1. [file elife-90603-fig1-figsupp2-data1.zip › Figure 1ΓÇöfigure supplement 2-source data 1/Figure 1ΓÇöfigure supplement 2A_rhodamine_annotated.tiff]

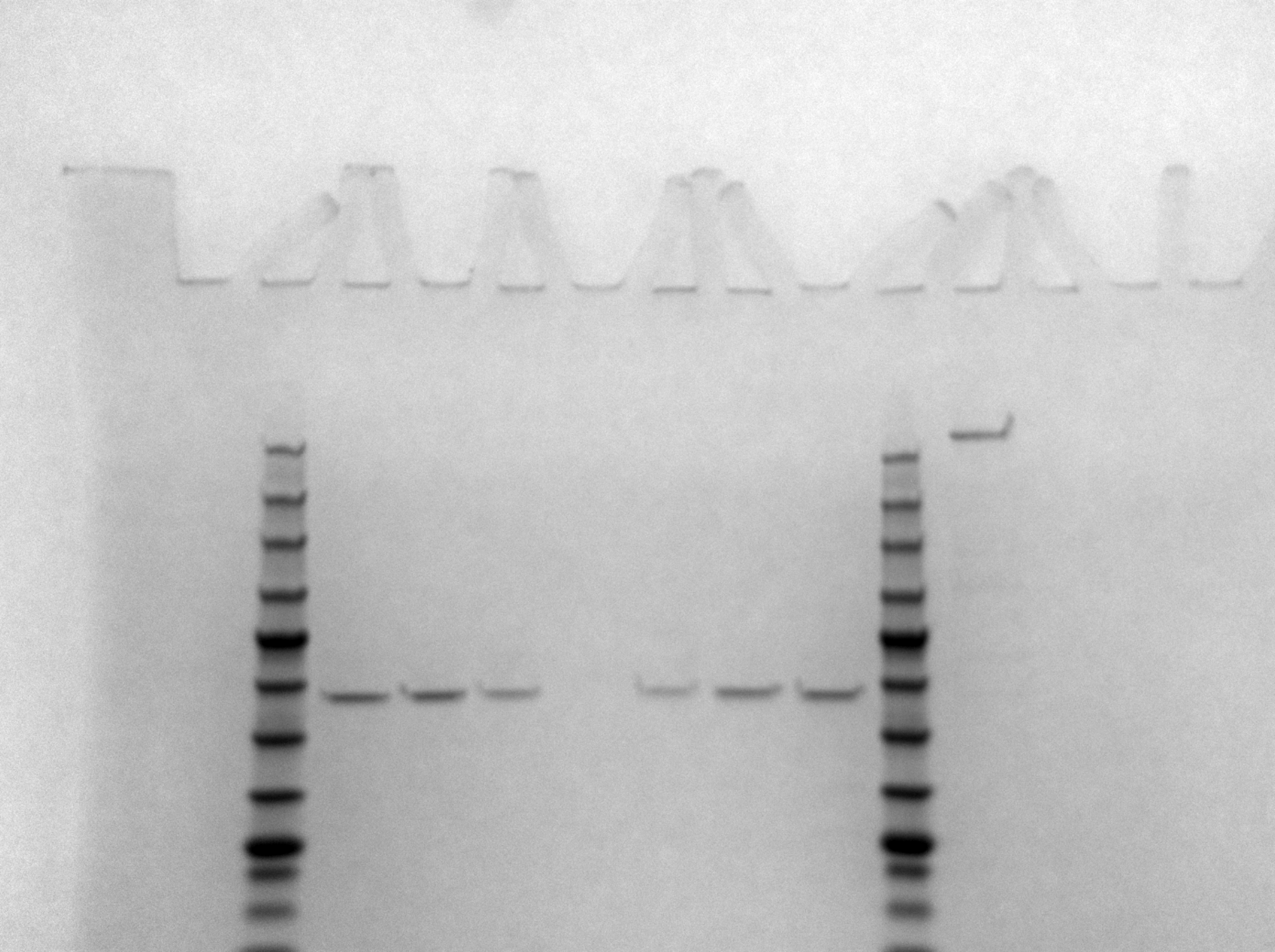

Supplement: Figure 1—figure supplement 2—source data 2. [file elife-90603-fig1-figsupp2-data2.zip › Figure 1ΓÇöfigure supplement 2-source data 2/Figure 1ΓÇöfigure supplement 2A_coomassie.tif]

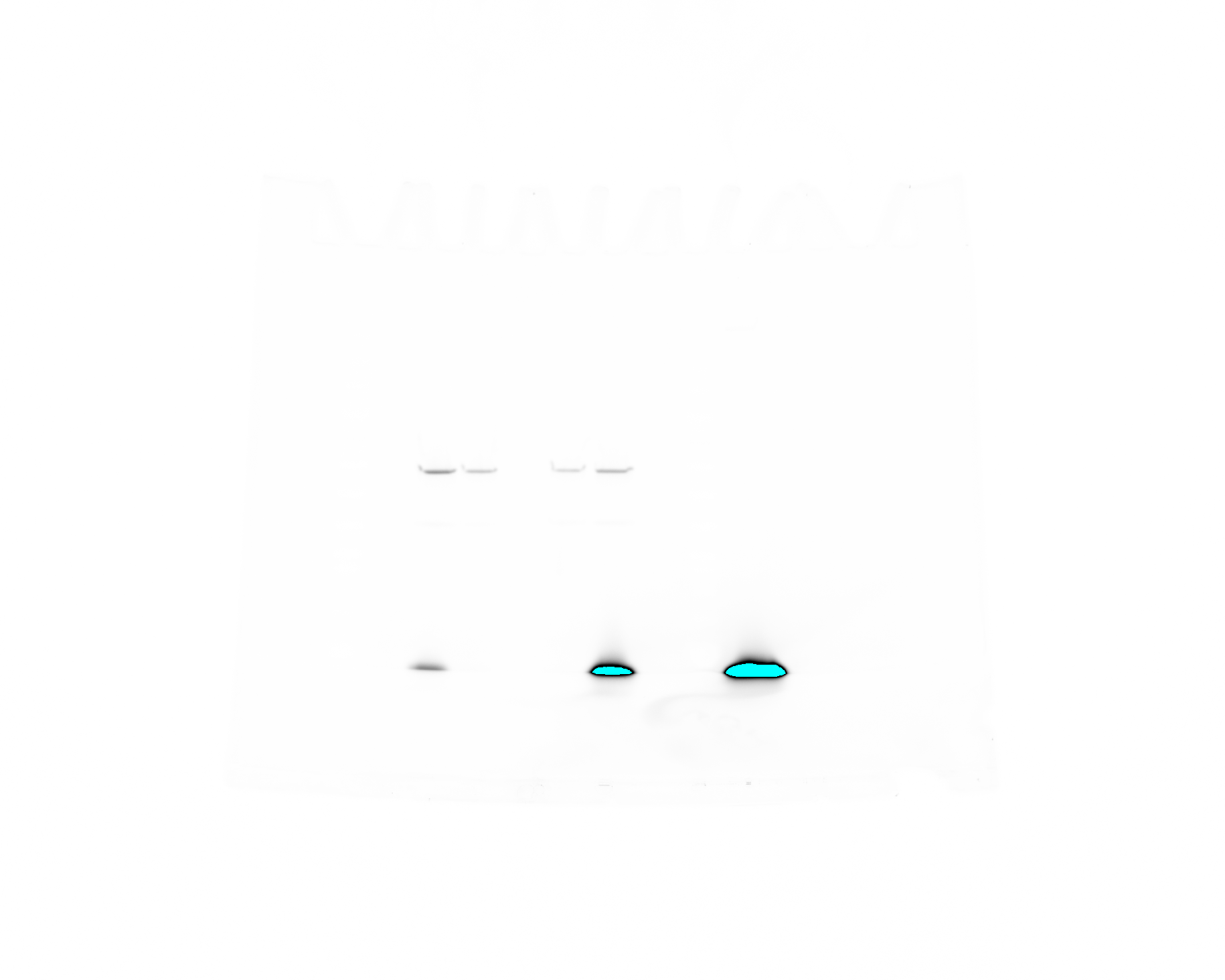

Supplement: Figure 1—figure supplement 2—source data 2. [file elife-90603-fig1-figsupp2-data2.zip › Figure 1ΓÇöfigure supplement 2-source data 2/Figure 1ΓÇöfigure supplement 2A_rhodamine.tif]

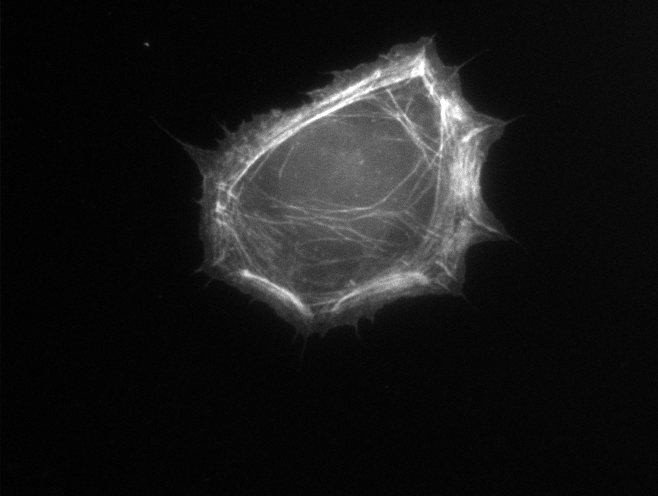

Supplement: Source code 1. [file elife-90603-code1.zip › Code/CellSegmentation/actin.tif]

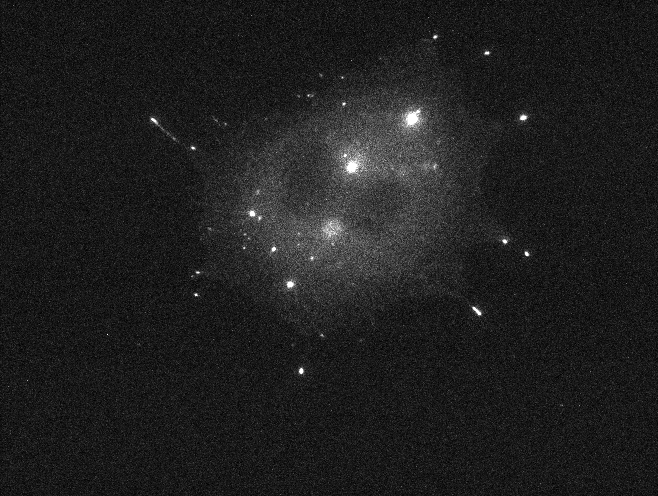

Supplement: Source code 1. [file elife-90603-code1.zip › Code/CellSegmentation/myo.tif]
